# ISL1 controls pancreatic alpha cell fate and beta cell maturation

**DOI:** 10.1186/s13578-023-01003-9

**Published:** 2023-03-10

**Authors:** Romana Bohuslavova, Valeria Fabriciova, Laura Lebrón-Mora, Jessica Malfatti, Ondrej Smolik, Lukas Valihrach, Sarka Benesova, Daniel Zucha, Zuzana Berkova, Frantisek Saudek, Sylvia M Evans, Gabriela Pavlinkova

**Affiliations:** 1 Laboratory of Molecular Pathogenetics, Institute of Biotechnology CAS, 25250 Vestec, Czechia; 2 Laboratory of Gene Expression, Institute of Biotechnology CAS, 25250 Vestec, Czechia; 3 Laboratory of Pancreatic Islets, Institute for Clinical and Experimental Medicine, 14021 Prague, Czechia; 4 Department of Pharmacology; Skaggs School of Pharmacy and Pharmaceutical Sciences, University of California at San Diego, La Jolla, CA USA

**Keywords:** Pancreatic endocrine cells, Epigenetic histone modification, Transcriptome, Pancreas development

## Abstract

**Background:**

Glucose homeostasis is dependent on functional pancreatic α and ß cells. The mechanisms underlying the generation and maturation of these endocrine cells remain unclear.

**Results:**

We unravel the molecular mode of action of ISL1 in controlling α cell fate and the formation of functional ß cells in the pancreas. By combining transgenic mouse models, transcriptomic and epigenomic profiling, we uncover that elimination of *Isl1* results in a diabetic phenotype with a complete loss of α cells, disrupted pancreatic islet architecture, downregulation of key ß-cell regulators and maturation markers of ß cells, and an enrichment in an intermediate endocrine progenitor transcriptomic profile.

**Conclusions:**

Mechanistically, apart from the altered transcriptome of pancreatic endocrine cells, *Isl1* elimination results in altered silencing H3K27me3 histone modifications in the promoter regions of genes that are essential for endocrine cell differentiation. Our results thus show that ISL1 transcriptionally and epigenetically controls α cell fate competence, and ß cell maturation, suggesting that ISL1 is a critical component for generating functional α and ß cells.

**Supplementary Information:**

The online version contains supplementary material available at 10.1186/s13578-023-01003-9.

## Background

Understanding the mechanisms that regulate generation and maintenance of pancreatic endocrine cells is critical for developing treatments for diabetes. Pancreatic endocrine hormone-secreting α (glucagon), β (insulin), δ (somatostatin), ε (ghrelin), and PP (pancreatic polypeptide) cells form the islets of Langerhans, which are essential for regulating glucose homeostasis.

The early organogenesis of the pancreas undergoes two main transitions (reviewed in [1–3]): the primary transition between E9.5-E12.5 in the mouse, when glucagon-expressing cells are formed preferentially, and the secondary transition at ~ E13.5-E15.5, when all types of endocrine cells are produced. It is not clear whether glucagon-expressing cells generated during the primary transition persist into adulthood [4]. During the secondary transition, endocrine progenitors have a higher propensity to differentiate into α cells at earlier stages, whereas at later stages they preferentially form β cells [5]. All hormone^+^ islet cell types in mice originate from Neurogenin 3 (NGN3) expressing endocrine progenitors [4–6]. NGN3^+^ endocrine progenitors proceed to differentiate via the activation of complex gene regulatory networks through multiple intermediate cell stages (reviewed in [7]).

Recent studies have shown that endocrine differentiation into distinct islet lineages is also regulated epigenetically [5, 8–12]. Epigenetic modifiers facilitate histone and nuclear DNA modifications that induce chromatin opening, and recruitment of additional transcription factors and other regulatory proteins that subsequently activate transcriptional programs of lineage specification and differentiation. For example, pancreatic β cells deficient in DNA methyltransferase 1 (*Dnmt1*) are reprogrammed to α cells via DNA hypomethylation of aristaless related homeobox (*Arx*) enhancers [13]. Conditional deletion of *Dnmt3a* mediated by Ins-Cre results in β-to-α-cell transdifferentiation in adult mice [14]. Deletion of *Kdm6b*, a histone demethylase for histone 3 lysine 27 trimethyl (H3K27me3), in endocrine progenitors results in abnormalities in the formation of islets, reduced overall endocrine mass, and a diabetic phenotype [11]. The presence of both repressive H3K27me3 and activating histone 3 lysine 4 trimethyl (H3K4me3) epigenetic marks of histone modifications in the promoter regions of regulatory genes represent a dynamic regulation of cell fate differentiation during endocrine pancreatic development [9].

ISL1, a LIM-homeodomain transcription factor, has a crucial role in neuronal, cardiac, and sensory development [15–18]. Apart from the direct transcriptional regulation of multiple downstream targets, emerging evidence indicates that ISL1 is an important factor in the epigenetic control of embryonic development, forming regulatory complexes involved in histone modifications [19, 20], regulating KDM6B demethylase activity [21], chromatin looping [22], and acting as a pioneer factor [23]. In the pancreas, ISL1 is expressed in all developing endocrine cells [5, 24, 25], and in adult α-, β-, PP-, and δ-islet cells [26, 27]. In adult β-cells, ISL1 interacts with neurogenic differentiation factor 1 (NEUROD1) to maintain insulin gene transcription activity [28], and furthermore, ISL1 alters the status of histone H3K4 and H3K27 methylation on the insulin promoter based on glucose concentrations [29]. During pancreas development*,* the first ISL1 expressing cells are detected in the dorsal pancreatic epithelium at E9.0 [24]. Germline knockout of the *Isl1* gene results in a complete loss of differentiating endocrine cells without affecting the expression domain of pancreatic and duodenal homeobox 1 (PDX1) in the dorsal pancreatic epithelium at E9.5 [24]. Embryonic arrest at E9.5 and lethality of *Isl1-null* mice [30] preclude an in-depth investigation of mechanisms by which ISL1 contributes to pancreatic endocrine development. Delayed conditional deletion of *Isl1* in *Pdx1*^*late*^*Cre;Isl1*^*f/f*^ during the secondary transition results in a severe hyperglycemia phenotype due to a significant reduction in insulin^+^, glucagon^+^, and somatostatin^+^ endocrine cells [31]. Although the cellular phenotype has been described previously [31], we still lack mechanistic insight into the critical role of ISL1 in endocrine pancreatic development and function. In particular, no investigation has been done of pancreatic endocrine development during the primary transition in the context of ISL1 deletion, nor ISL1 function during the transition from early undifferentiated cell types to mature adult-like cell states. Thus, despite the established critical role of ISL1 in endocrine pancreatic development and function, much remains to be discovered to understand its molecular mode of action.

We created an early conditional deletion of *Isl1* (*Isl1CKO*) that disrupts the primary transition of pancreas endocrine development. We aimed to determine the role of ISL1 in α- and β- cell differentiation and maturation using transcriptome-wide gene expression profiling. Critically, our molecular analyses of *Isl1CKO* endocrine cells show a shift in the transcriptomic signature towards intermediate progenitor states, loss of α-cell differentiation, and changes in molecular programs driving the formation of mature β cells. Additionally, elimination of *Isl1* affected repressive H3K27me3 and activating H3K4me3 modification patterns in promoter regions of differentially expressed genes in pancreatic endocrine cells. Thus, these findings provide mechanistic insight into the role of ISL1 in transcriptional and epigenetic regulatory networks orchestrating the proper development and maturation of pancreatic islet α and β cells.

## Results

### Elimination of *Isl1* results in a diabetic phenotype with impaired molecular characteristics of islets of Langerhans

**Fig. 1:**
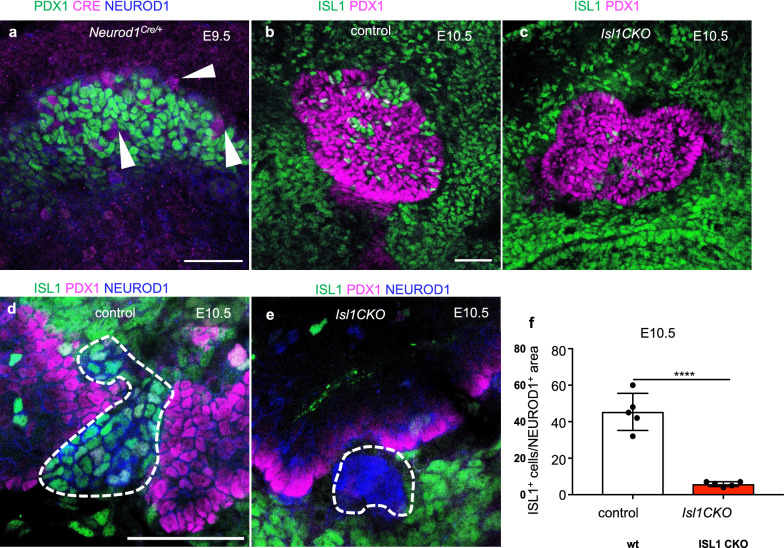
Efficient deletion of ISL1 in the *Isl1CKO* pancreas during the primary transition. **a** Representative whole-mount immunolabeling of the dorsal pancreas shows that NEUROD1 matches Neurod1^Cre^ expression visualized by anti-CRE antibody in the PDX1^+^ pancreatic domain (arrowheads indicate cells co-expressing CRE and NEUROD1). **b**, **c** A reduced number of ISL1^+^ cells shown in the *Isl1CKO* dorsal pancreatic epithelium delineated by the expression of PDX1 (whole mounts). **d–f** Higher-magnification images show significantly reduced number of cells expressing ISL1 in NEUROD1^+^ area in the *Isl1CKO* dorsal pancreas compared to littermate controls. Data are presented as mean ± SD (n = 5 pancreases per genotype), unpaired *t*-test (****P < 0.0001). Scale bars: 50 μm

To determine the functional requirements of ISL1 for the development of endocrine cells in the pancreas, we generated novel *Isl1* conditional knockout mice by crossing *Neurod1*^*Cre/*+^ mice [32] with floxed *Isl1* (*Isl1*^*f/f*^*)* [15]. Neurod1^Cre^ activity was detected at the initiation of the first transformation of endocrine cell formation in pancreas development at E9.5 and corresponded to the expression pattern of NEUROD1 (Fig. 1a). Accordingly, ISL1 expression was apparently reduced in the dorsal pancreas of *Isl1CKO* compared to control littermates at E10.5 (Fig. 1b, c). Noticeably, ISL1-positive mesenchymal cells surrounded the PDX1^+^ pancreas, as previously described [24]. Analyses of ISL1 expression confirmed efficient Neurod1^Cre^-mediated *Isl1* elimination, as 87% of NEUROD1^+^ progenitors in the dorsal pancreatic epithelium of *Isl1CKO* did not express ISL1 compared to controls at E10.5 (Fig. 1d–f). Virtually no expression of ISL1 was detected later in endocrine cells of the developing pancreas (Additional file 1: Fig. S1). Thus, the use of the Neurod1^Cre^ driver line led to the efficient and earlier elimination of ISL1 than observed previously in the *Pdx1*^*late*^*Cre;Isl1*^*f/f*^ model with reported elimination of ISL1 protein at E13.5 [31, 33].

**Fig. 2:**
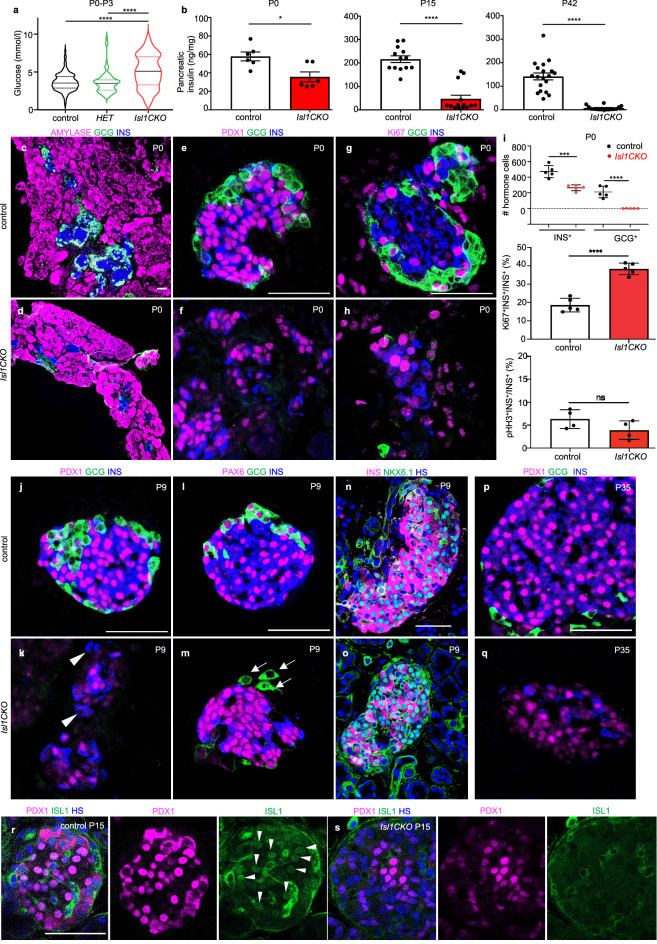
Physiological changes and molecular abnormalities in islets of Langerhans associated with diabetic phenotype of *Isl1CKO. ***a** Blood glucose levels of *Isl1CKO* (n = 59 pups), heterozygous (*HET*, *NeuroD1*^*Cre*^*/Isl1*^*f/*+^; n = 77 pups), and controls (n = 138 pups) fed ad libitum after birth at P0-P3. Violin plots indicate median (middle line), 25th, and 75th percentile (dotted lines). Data were analyzed by one-way ANOVA with Tukey’s multiple comparisons test (****P < 0.0001). **b** Total pancreatic insulin content per pancreatic tissue (ng/mg) at P0 (n = 6 pancreases per genotype), P15 (n = 13 pancreases per control, 14 pancreases per *Isl1CKO*), and in the adult mice (n = 19 pancreases per control, n = 16 pancreases per *Isl1CKO*) fed ad libitum. Data are presented as mean ± SEM, Student’s *t* test (*P = 0.017, ****P < 0.0001). **c-f** Representative sections from the control and *Isl1CKO* pancreas immunostained for glucagon (GCG), insulin (INS), PDX1 (marker of β cells), or alpha amylase (marker for exocrine cells) at P0 demonstrate reduced endocrine tissue and abnormalities in the formation of pancreatic islets in *Isl1CKO* with lower production of INS, missing GCG^+^ cells, and reduced expression of PDX1. **g**, **h** Immunolabeling for proliferating cell nuclear antigen Ki67 (red) shows proliferating GCG^+^ and INS^+^ cells in the islets of the *Isl1CKO* and control pancreas at P0. **i** Total number of INS^+^ and GCG^+^ cells in 80-μm sections of the pancreas (n = 5 pancreases per genotype), and the percentage of INS^+^ cells expressing Ki67 (n = 5 mice per genotype) and phosphorylated histone H3 (pHH3; n = 4 mice per genotype). See also Additional file 1: Fig. S3. Data are presented as mean ± SD, unpaired *t* test (****P < 0.0001, ***P < 0.001, ns = not significant). **j-o** Immunostaining of α- and β-cell markers (PDX1, INS, GCG, PAX6, and NKX6.1) in the pancreatic sections at P9 shows abnormalities in β cells, including reduced production of INS, variable expression levels of PDX1 with some cells expressing INS but not PDX1 (arrowheads in **k** indicate INS^+^ cells without PDX1 expression). GCG producing α cells are lost in the *Isl1CKO* islets of Langerhans. Arrows in **m** indicate the unusual GCG^+^ cells with a missing expression of PAX6, a marker of both β and α cells. **p**, **q** At P35, compare to the control pancreatic islets, the expression of INS and PDX1 is reduced, and GCG^+^ producing cells are missing in the *Isl1CKO* islets. **r**, **s** Representative confocal microscopy images of double staining with anti-PDX1 and anti-ISL1 antibodies show that the expression of ISL1 is not detected in nuclei of endocrine cells of pancreatic islets of *Isl1CKO* in contrast to the control. Arrowheads indicate nuclei with ISL1 and PDX1 co-expression in the control pancreatic islet. Note reduced PDX1 expression in *Isl1CKO*. Scale bars: 50 μm. HS, Hoechst nuclear staining

*Isl1CKO* mice demonstrated a severe diabetic phenotype with significant neonatal hyperglycemia that worsened with age (Fig. 2a; Additional file 1: Fig. S2), consistent with earlier findings using delayed conditional deletion of *Isl1* in *Pdx1*^*late*^*Cre;Isl1*^*f/f*^ [31]. None of the *Isl1CKO* mice survived past 3 months of age. Blood glucose concentration was higher and more variable in neonatal *Isl1CKO* than in heterozygous and control littermates (Fig. 2a). Accordingly, total pancreatic insulin content was significantly reduced already at P0 and more than 25 times lower in adult pancreas of *Isl1CKO* mice fed ad libitum (3.509 ± 0.678 ng/mg, n = 19) compared to controls (88.29 ± 6.539 ng/mg, n = 12; Fig. 2b). Both female and male *Isl1CKO* mice fed ad libitum showed increased blood glucose levels during postnatal development before weaning compared to controls and *Isl1* heterozygous mutants (Additional file 1: Fig. S2a). Of the 40 *Isl1CKO* mice measured, approximately 50% had unmeasurable fasting blood glucose levels (> 35 mmol/L). Glucose tolerance tests (GTTs) confirmed that both male and female heterozygous *Neurod1*^*Cre/*+^*; Isl1*^*f/*+^ mice were comparable to controls (Additional file 1: Fig. S2c), but we were unable to perform GTTs by intraperitoneal injection of glucose (2 g/kg body weight) in *Isl1CKO*. Although we selected *Isl1CKO* mice with low hyperglycemia, the mutants died after administration of exogenous glucose, indicating a severe inability to maintain an insulin secretion response when challenged with glucose.

Next, we investigated the formation and structure of islets of Langerhans using anti-insulin and glucagon as markers for β and α cells, respectively. At P0, the islets of Langerhans of *Isl1CKOs* contained only β cells expressing insulin and PDX1, a marker of differentiated β cells (Fig. 2c–f). Interestingly, immunostaining for proliferating cell nuclear antigen Ki67 revealed an increased number of β cells positive for Ki67 in the *Isl1CKO* pancreas at P0 (Fig. 2g–i). As Ki67 marks all cells engaged in the cell cycle, we also used the marker of cellular mitosis, phosphorylated histone H3 (pHH3) [34]. Quantification of the percentage of β (insulin^+^) cells that were pHH3 positive indicated similar mitotic activity in the *Isl1CKO* and control pancreases (Fig. 2i, Additional file 1: Fig. S3). A high Ki67 index may reflect a lengthened cell cycle or cell-cycle arrest of *Isl1CKO* β cells.

At P9, when a mature functional glucose-stimulated-insulin-secretion phenotype of β-cells is acquired [35], instead of β cells co-expressing insulin and PDX1 as shown in controls (Fig. 2j), expression of PDX1 was undetectable in many insulin^+^ cells in *Isl1CKO* (arrowheads in Fig. 2k). In contrast to PDX1, the expression of paired box 6 (PAX6), which is expressed in both glucagon and insulin-expressing cells in controls, seemed unaffected in *Isl1CKO* β cells (Fig. 2l, m). Note the unusual glucagon^+^ cells without the expression of PAX6 (arrows in Fig. 2m), indicating abnormalities in the differentiation of these cells. Immunolabeling for the β-cell programing factor NK 6 homeobox1 (NKX6.1) demonstrated that many NKX6.1^+^ cells did not express insulin compared to controls (Fig. 2n, o). Profound diminished PDX1 levels were found in adult *Isl1CKO* pancreas (P35), correlating with a reduced number of insulin-producing cells in the islets of Langerhans and severe diabetic phenotype of *Isl1CKO* mice (Fig. 2p, q). Diminished PDX1 expression reflected essentially complete elimination of ISL1 in *Isl1CKO* pancreatic islets (Fig. 2r, s).

**Fig. 3:**
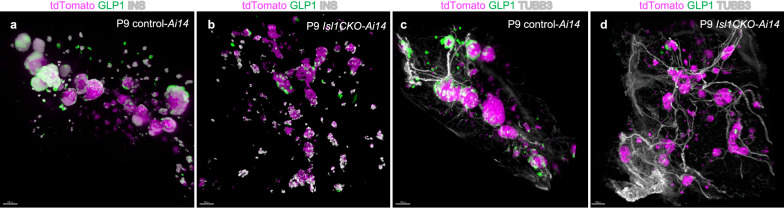
Distribution and formation of islets of Langerhans in the microenvironment of the pancreas. Microdissected pancreases of tdTomato reporter control-*Ai14* and *Isl1CKO*-*Ai14* mice were cleared (CUBIC protocol), immunolabeled, imaged, and reconstructed in 3D using light-sheet fluorescence microscopy (LFSM; see Additional files 2, 3, 4, 5: Videos S1–S4). LFSM images depict the distribution and formation of islets in the anatomical microenvironment of the pancreas at P9, showing tdTomato^+^ endocrine cell population and INS producing β cells together with anti-GLP1 labeled α cells in (**a**, **b**) or with neuronal fibers labeled by anti-TUBB3 in (**c**, **d**). Scale bars: 100 μm

We applied light sheet fluorescence microscopy (LSFM) to uncover the formation, and spatial distribution of the islets of Langerhans in the 3D tissue microenvironment of the pancreas (Fig. 3a–d, Additional files 2, 3, 4, 5: Videos S1-S4). Endocrine cells were visualized by combinations of genetic labeling using tdTomato reporter expression and immunolabeling of α cells with anti-GLP1 (glucagon like peptide 1) and β-cells with anti-insulin (Fig. 3a, b). In the second preparation, tdTomato^+^ endocrine cells were co-labeled with anti-GLP1 (α cells) and anti-TUBB3 depicted innervation in the pancreas (Fig. 3c, d). Endocrine cells were scattered, forming smaller cell clumps without α cells at the periphery in the *Isl1CKO* pancreas compared to the characteristic islet structure of the control pancreas. In line with abnormalities in islet formation, the total number of isolated islets from the adult *Isl1CKO* pancreas was severely reduced compared to controls (11 ± 10 islets/*Isl1CKO* pancreas, n = 8 *vs.* 273 ± 77 islets/control pancreas, n = 6, *P* < 0.0001). Taken together, these changes indicated a loss of α cells, abnormalities in PDX1 expression in β cells, production of insulin, and formation of the islets of Langerhans in *Isl1CKO*.

### Elimination of *Isl1* alters the α-cell differentiation program during the primary transition of pancreas development

**Fig. 4:**
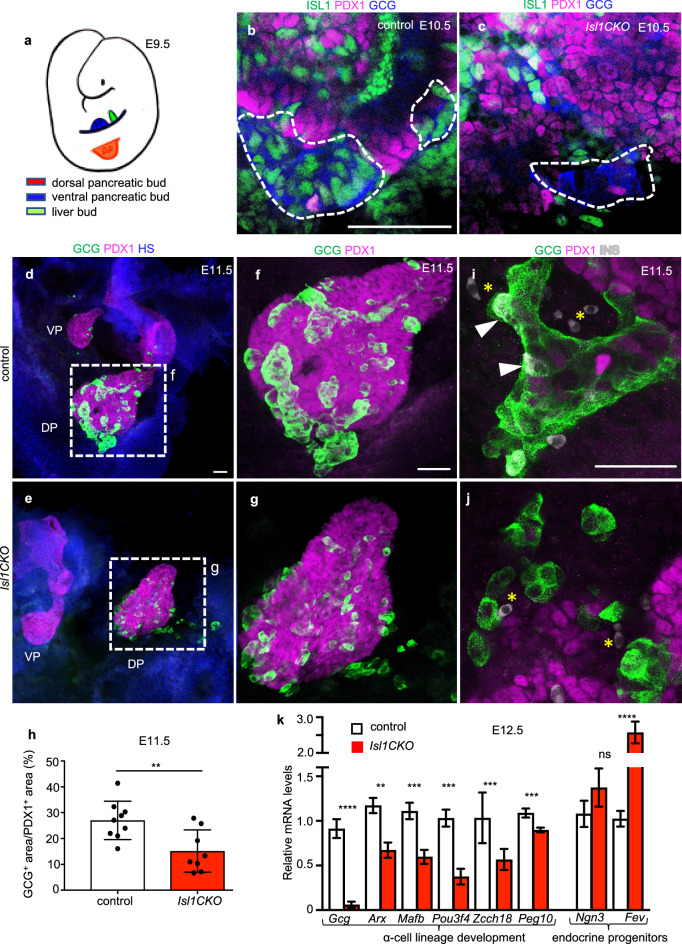
Aberrant α-cell lineage development during primary transition in the *Isl1CKO* pancreas.** a** Schematic presentation of the pancreas formation, which begins with the independent budding of the dorsal and ventral buds at the posterior region of the foregut. These two buds, surrounded by the mesenchyme, eventually fuse after rotation of the gut to form the pancreatic endoderm. **b**, **c** Representative higher-magnification images of whole-mount immunolabeling show a loss of ISL1 and glucagon (GCG) expressing cells in the dorsal pancreas of *Isl1CKO* at E10.5. **d-g** Whole-mount immunolabeling shows the formation of GCG^+^ clusters in the dorsal (DP) and ventral pancreatic buds (VP) at E11.5. Higher-magnification images show DP with GCG^+^ clusters. **h** Quantification of the GCG^+^ area in the PDX1^+^ domain shows the reduced size of GCG^+^ clusters in the dorsal pancreas of *Isl1CKO* (n = 8 pancreases) compared to controls (n = 9 pancreases). Data are presented as mean ± SD, Unpaired *t*-test, **P = 0.0069). **i, j** Representative images of whole-mount immunolabeling of the pancreas show endocrine cells co-expressing GCG and insulin (INS) in the control (arrowheads) but not in the *Isl1CKO* pancreas (asterisks indicate autofluorescent red blood cells). **k** Quantitative RT-PCR analyses of mRNA levels of *Gcg* and selected transcription factors in the E12.5 pancreas. Data are presented as mean ± SEM (n = 8 pancreases per genotype), Unpaired *t*-test (****P < 0.0001, ***P < 0.001, **P < 0.01, ns = not significant). Scale bars: 50 μm

The primary transition of mouse pancreas development is initiated by expression of the transcription factor PDX1 that specifies multipotent pancreatic progenitors, and evagination of a dorsal pancreatic bud from the foregut endoderm around E9.0 [1] (Fig. 4a). The primary transition of endocrine cell formation mainly generates glucagon-expressing α cells. To investigate changes associated with *Isl1* deletion during the primary transition, we evaluated the presence of glucagon-expressing cells in the dorsal pancreatic bud, as their appearance represents the first sign of endocrine cell differentiation [4]. Glucagon-producing cells at the periphery of the dorsal pancreatic bud of *Isl1CKO* did not express ISL1 (Fig. 4b, c). Overall, the generation of glucagon^+^ cells and formation of glucagon^+^ clusters during the first transition was significantly reduced by 50% in the dorsal pancreas of *Isl1CKO* compared to littermate controls (Fig. 4d–h). Additionally, a transient population of developing endocrine cells co-producing glucagon and insulin was diminished in *Isl1CKO*, indicating changes in the composition of glucagon-producing subpopulations (Fig. 4i, j). Defects in developmental programs during early pancreatic organogenesis were further confirmed by qPCR at E12.5 (Fig. 4k). Expression of key genes encoding differentiation regulators of the α-cell lineage, such as *Arx1*, *MafB*, *Peg10*, and *Pou3f4*, were significantly reduced in developing *Isl1CKO* pancreas at E12.5. Interestingly, expression of the E26 transformation-specific transcription factor, *Fev,* was increased. A pancreas lineage study suggested that *Fev*^+^ cells represent an intermediate endocrine progenitor state following *Ngn3* expression [36]. Consistent with the *Fev*^+^ endocrine progenitor expression profile [36, 37], we found no changes in *Ngn3* levels in the developing pancreas of *Isl1CKO* (Fig. 4k). These results suggested that elimination of *Isl1* abolished α-cell lineage development during the primary transition.

### The α-cell population disappears during the secondary transition and ß-cell proliferation is reduced in *Isl1CKO* pancreas development

**Fig. 5:**
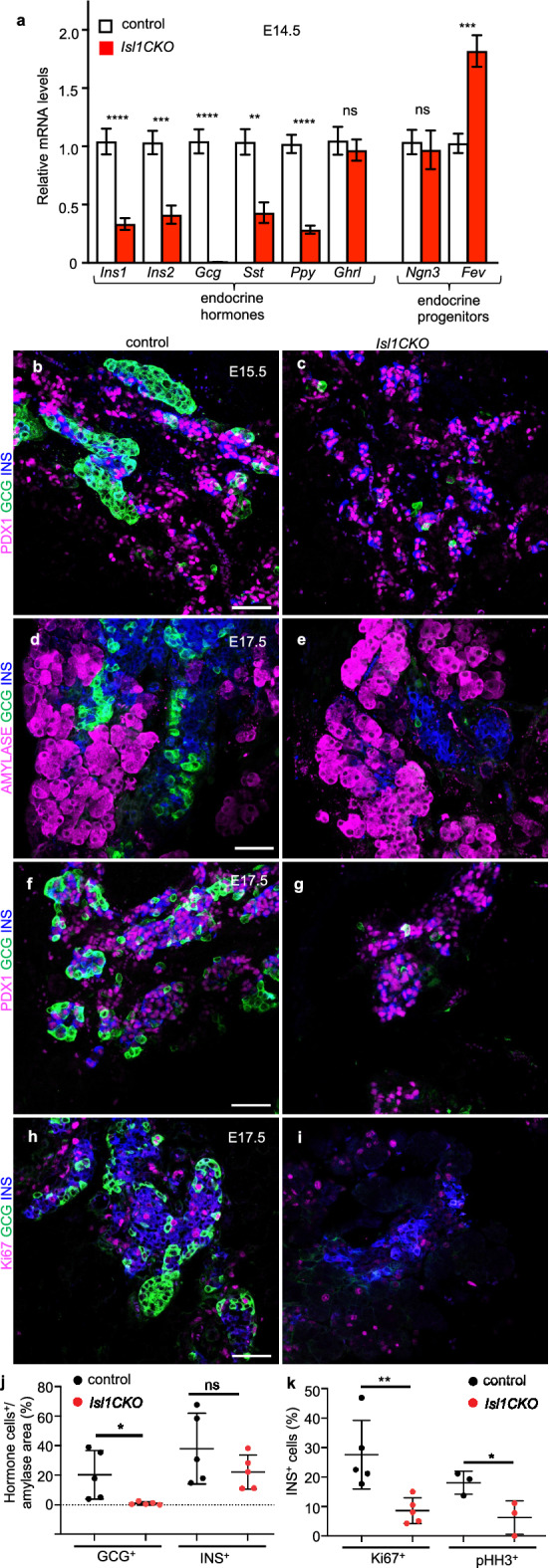
Attenuated expression of insulin and a loss of glucagon producing cells during the secondary transition of pancreas development in *Isl1CKO*.** a** Quantitative RT-PCR analyses show reduced mRNA levels of endocrine hormones and increased expression of the transcription factor *Fev* in the E14.5 pancreas of *Isl1CKO* compared to controls. Data are presented as mean ± SEM (n = 8 pancreases per control, n = 7 pancreases per *Isl1CKO*), Unpaired *t*-test (****P < 0.0001, ***P < 0.001, **P < 0.01). **b-g** Representative sections from the control and *Isl1CKO* pancreas immunostained for glucagon (GCG), insulin (INS), PDX1 (differentiation marker of β cells), or alpha amylase (marker for exocrine cells) demonstrate loss of endocrine α cells (glucagon, GCG) in *Isl1CKO*. **h**, **i** Representative sections immunostained for proliferating cell nuclear antigen Ki67 in endocrine α (GCG) and β cells (INS). **j** Relative quantification of GCG^+^ and INS^+^ cells per α-amylase^+^ area (marker of exocrine tissue; n = 5 pancreases per genotype), and **k** the percentage of INS^+^ cells expressing Ki67 (n = 5 pancreases per genotype) and phosphorylated histone H3 (pHH3; n = 3 pancreases per genotype) per total number of INS^+^ cells is decreased in the *Isl1CKO* pancreas compared to littermate controls at E17.5. See also Additional file 1: Fig. S3. Data are presented as mean ± SD. Unpaired *t*-test (**P < 0.01, *P < 0.05, ns = not significant). Scale bars: 50 μm

The trend of increased expression of *Fev* continued in the developing *Isl1CKO* pancreas at E14.5, during the secondary transition (Fig. 5a). The secondary transition represents morphogenetic events resulting in a branched structure of the epithelium containing endocrine progenitors that differentiate into different endocrine cells [3]. The early elimination of *Isl1* in *Isl1CKO* resulted in a faster onset of changes in endocrine development than observed previously after *Isl1* deletion using a delayed *Pdx1*^*late*^*Cre;Isl1*^*f/f*^ model [31]. We confirmed there was a significant reduction of insulin, somatostatin, and pancreatic polypeptide mRNA in the developing *Isl1CKO* pancreas as early as E14.5 (Fig. 5a). We found essentially no glucagon mRNA expression in the E14.5 pancreas of *Isl1CKO* (Fig. 5a), indicating that the use of the Neurod1^Cre^ driver line led to an earlier elimination of glucagon production than observed previously [31]. In line with changes in mRNA expression, a near complete loss of glucagon^+^ cells was found in *Isl1CKO* pancreas at E15.5 (Fig. 5b, c). ß-cell proliferation was already significantly diminished in *Isl1CKO* compared to littermate controls at E17.5 (Fig. 5d–i), in contrast to the previously reported reduced proliferation of ß cells in *Pdx1*^*late*^*Cre;Isl1*^*f/f*^ at P6 [31]. The percentage of ß cells expressing the proliferation marker Ki67 was decreased nearly threefold in *Isl1CKO* pancreas compared to control littermates at E17.5. Similarly, ß cells in the *Isl1CKO* pancreas proliferated less as shown by pHH3 staining, the marker of cellular mitosis. These data indicate that ISL1 is important for the induction of the expansion of the endocrine ß-cell population during pancreas development. Notably, expression of PDX1 was comparable between control and *Isl1CKO* endocrine cells at E17.5.

### Elimination of *Isl1* results in a shift of transcriptomic signatures characterizing endocrine cell populations in the E14.5 pancreas

**Fig. 6:**
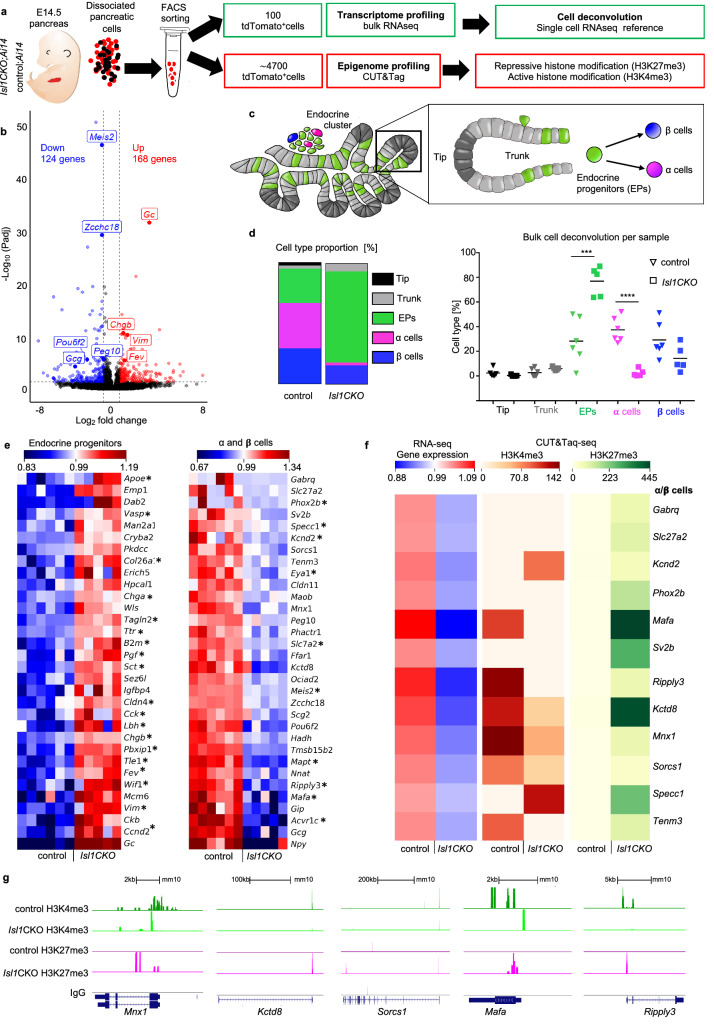
*Isl1* elimination induces transcriptomic and epigenetic changes altering the composition of the endocrine cell population in the developing pancreas.** a** Overview of study design. **b** Volcano plot showing differentially expressed genes between FACS-sorted pancreatic endocrine cells from *Isl1CKO* (n = 5) and control E14.5 embryos (n = 6) identified by RNA sequencing (Padj < 0.05 displayed as –log_10_ and log_2_ fold change − 1 and 0.585). Thresholds are indicated by dotted lines. Complete list of differentially expressed genes in Supplementary Dataset 1a. **c** A simplified schematic overview of the different pancreatic cell types represented in single cell RNA-seq [39] used as a reference for the cell type deconvolution of our bulk cell RNA-seq data. **d** The deconvolved cell type proportions in E14.5 endocrine population from our bulk RNA-seq data. The proportion of major cell types is shown as an average and per individual samples. Data are presented as mean ± SD, Unpaired *t*-test (****P < 0.0001, ***P = 0.0007). See also Additional file 1: Fig. S4. **e** Heatmaps represent the top 30 enriched genes in endocrine progenitors, and in α and β cells in *Isl1CKO* and control samples based on the transcriptome analyses (*indicates genes with ISL1 binding sites at their promoter regions identified by the ISL1 CUT&Tag-seq, see page 13). Complete list of genes in Supplementary Dataset 1b and 1c, and S2. **f** Heatmap representation of expression profiles of differentially expressed genes that have differences in H3K4me3 or H3K27me3 enrichment in their promoter regions (± 3 kb from the TSS) between control and *Isl1CKO* cells. Scale bar for RNA-seq data represents average values calculated from the ratio of rlog of the sample to average rlog of the row (all samples per gene). Scale bars for CUT&Taq-seq data represent average counts per million (cpm; > 25 cpm) per each group. **g** Genome track view of representative gene loci showing H3K4me3 (green), H3K27me3 (magenta), and negative antibody control IgG (grey) normalized read peaks. EPs, endocrine progenitors

To gain insight into molecular mechanisms underlying ISL1 function, we performed RNA-seq analyses of pancreatic endocrine cells during the secondary transition at E14.5, as E14.5 endocrine progenitors have a higher propensity to form α cells [5]. We opted to use Bulk-RNA sequencing to obtain sequencing depth and high-quality data [38]. Pancreases were dissected, dissociated into single cells, and 100 fluorescent tdTomato^+^ cells were isolated via fluorescence-activated cell sorting (FACS) per each biological replicate. We used a Rosa26-tdTomato reporter mouse line to genetically label *Neurod1*^*Cre*^*,* expressing endocrine cells in the *Isl1CKO* (genotype: *Isl1*^*f/f*^*; Neurod1*^*Cre*^*; TomatoAi14*; n = 5) and control pancreas (genotype: *Isl1*^*f/*+^*; Neurod1*^*Cre*^*; TomatoAi14*; n = 6) (experimental design in Fig. 6a). Compared to controls, 292 protein-coding genes were differentially expressed in *Isl1CKO* pancreatic endocrine cells (adjusted P-value, P_adj_ < 0.05, fold change > 1.5 and < 0.5 cut-off values; Fig. 6b and Additional file 6: Dataset S1a). Functional profiling revealed that the top gene clusters for down-regulated genes were related to highly enriched biological pathways and specific gene ontology (GO) term categories associated with synthesis, secretion, and inactivation of the incretion, GLP1, glucose-dependent insulinotropic polypeptide (GIP), glucagon receptor binding, and peptide hormone secretion (Additional file 1: Fig. S4a). We found a significant reduction of hallmark genes associated with the α-cell lineage in *Isl1CKO* endocrine cells, including *Peg10*, *Pou6f2*, and *Gcg* [5, 12, 38].

Using single-cell transcriptomic profiling data of cells in embryonic mouse pancreas [39] as a reference for cell type deconvolution, we estimated the proportion of cell types in our bulk RNA-seq samples [40]. Single cell transcriptomic signatures of five major cell types in the developing pancreas, e.g. endocrine progenitors, epithelial tip, trunk epithelium, ß and α cells, were used for the deconvolution of our bulk expression data (Fig. 6c, d, and Additional file 1: Fig. S4b, c). A significantly higher proportion of endocrine progenitors marked by high *Fev* expression was found in *Isl1CKOs* with enriched genes defining intermediate states of endocrine differentiation, such as *Fev, Chgb, Chga, Vim,* and *Cldn4* (Fig. 6d, e; Additional file 6: Dataset S1b) [5, 36, 39]. Concurrent with a deficiency in the generation of α cells in *Isl1CKOs*, the proportion of α cells was substantially decreased (Fig. 6d) together with a reduced expression of genes associated with the α-cell lineage, including *Gcg, Peg10*, *Pou6f2, Meis2, Ripply3,* and *Zcchc18* (Fig. 6e; Additional file 6: Dataset S1b) [5, 12, 38]. Interestingly, no α cells of a late differentiation stage (cluster 8) were found in *Isl1CKO* compared to control cells (Additional file 1: Fig. S4c), indicating differentiation arrest in the α-cell lineage. Motif enrichment transcription factor target analysis [41] showed that ISL1 and FEV were the top ranking transcription factors associated with differentially expressed genes in *Isl1CKO* together with transcription factors essential for endocrine specification, regulatory factor X6 (RFX6) [42], and paternally-expressed gene 3, PEG3 (also known as PW1) [43], suggesting a shift in the transcriptomic signature of *Isl1CKO* endocrine cells towards earlier progenitor states (Additional file 6: Dataset S1c).

### H3K4me3 and H3K27me3 patterns are changed at promoter regions in *Isl1CKO*

Since our data demonstrated that the deletion of *Isl1* resulted in a loss of α cells and enrichment of endocrine progenitor populations, we then asked whether *Isl1* elimination affected the epigenetic basis of endocrine cell identity in the pancreas. We chose to investigate histone H3 modifications, H3K4me3, a marker of active transcription and H3K27me3, a marker of transcriptional silencing, because of their roles in the regulation of transcription and cell-fate determination [9, 44]. Bivalently H3K4me3 and H3K27me3 marked promoters are associated with transcriptionally inactive genes or genes expressed at very low levels [45]. The presence of both marks keeps genes at a poised state enabling them to be rapidly activated, particularly during embryogenesis and differentiation [44]. We performed chromatin profiling of H3K4me3 and H3K27me3 modifications on FACS-sorted tdTomato^+^ cells from the E14.5 pancreas Cleavage Under Targets and Tagmentation sequencing (CUT&Tag-seq) [46] (experimental design in Fig. 6a). Genome-wide H3K4me3 or H3K27me3 marks were distributed similarly in control and *Isl1CKO* endocrine cells with the majority of H3K4me3 deposition at gene promoter regions, while H3K27me3 peaks were distributed along the promoter, gene body and intergenic regions (Additional file 1: Fig. S4d, e). To correlate H3K4me3 and H3K27me3 modifications with gene expression changes, we focused only on histone modifications at the promoter regions of differentially expressed genes identified in our RNA-seq (P_adj_ < 0.05 and Fold change 50%). We compared the normalized read counts of H3K4me3 or H3K27me3 to identify a variable H3 methylation pattern at promoter regions between control and *Isl1CKO* cells (Fig. 6f, Additional file 1: Fig. S4f). In *Isl1CKO* endocrine cells, downregulated regulatory genes, such as *Mafa, Ripply3, Sorcs1,* and *Mnx1*, were marked by a suppressive H3K27me3 methylation signature in contrast to control cells (Fig. 6g). These downregulated genes were associated with the α-and β-cell lineages (Fig. 6e). Our data confirmed that histone methylation patterns were affected in the absence of *Isl1*, contributing to abnormalities in the regulation of endocrine differentiation in the embryonic pancreas.

### Elimination of *Isl1* results in downregulation of maturation markers of β cells in the *Isl1CKO* pancreas at P9

**Fig. 7:**
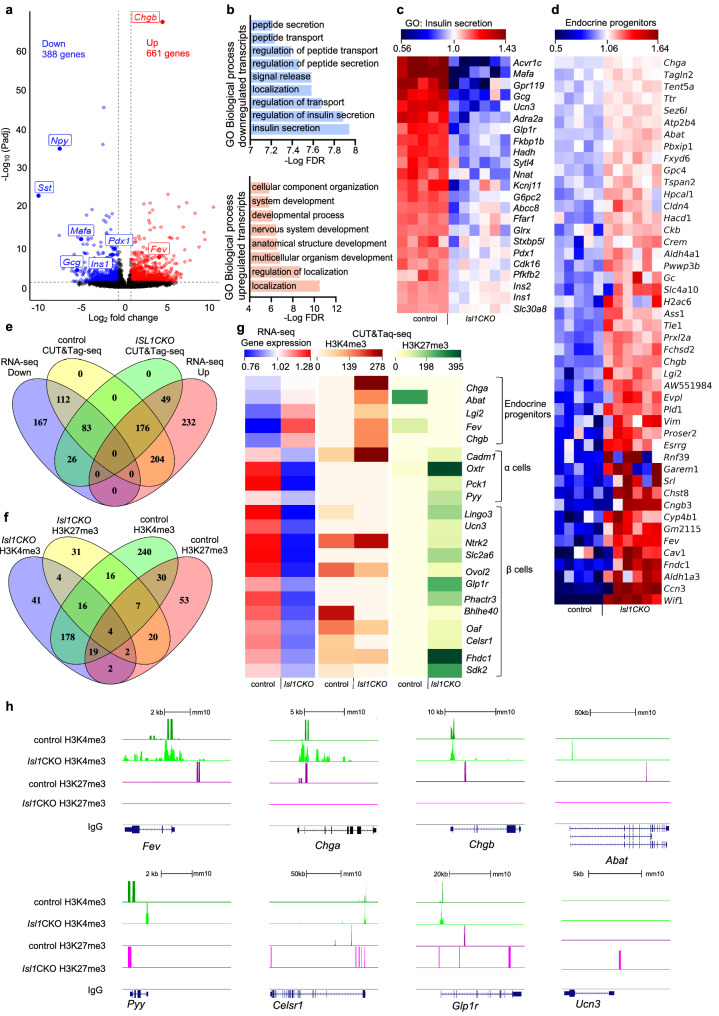
ISL1 modulates expression profiles of endocrine cells in the P9 pancreas.** a** Volcano plot shows differentially expressed genes (Padj < 0.05 (displayed as –log_10_) and log_2_ fold change − 1 and 0.585) identified by RNA sequencing in 100 FACS-sorted endocrine cells from the P9 pancreas of *Isl1CKO* (n = 6) in comparison to the control (n = 5). Complete list of genes in Supplementary Dataset 1d. **b** The most enriched Gene Ontology (GO) biological processes for downregulated and upregulated genes identified by RNA-seq. Complete list of genes for the GO terms in Supplementary Dataset 1e. **c** Heatmap of insulin secretion genes from the GO analysis shows expression levels for each sample. **d** Heatmap shows the top upregulated genes in P9 endocrine cells of *Isl1CKO* that were found enriched in E14.5 endocrine progenitors of *Isl1CKO* based on deconvolution analyses (Supplementary Dataset 1f). **e** Venn diagram representing the overlap of genes with differential expression (RNA-seq) and genes with H3K4me3 and/or H3K27me3 marks (CUT&Tag-seq) in their promoter regions (± 3 kb from the TSS) in control and *Isl1CKO* endocrine cells at P9. **f** Overlap between H3K4me3 and H3K27me3 modifications in the promoter regions of differentially expressed genes in control and *Isl1CKO* endocrine cells. **g** Heatmap shows the selection of differentially expressed genes identified by RNA-seq (average expression level per control and *Isl1CKO*) correlating to differences in H3K4me3 and H3K27me3 modification patterns at their promoter regions (± 3 kb from the TSS) between control and *Isl1CKO* pancreatic endocrine cells at P9. **h** Genome track view of representative gene loci showing H3K4me3 (green), H3K27me3 (magenta), and negative antibody control IgG (grey) normalized read peaks based on CUT&Tag-seq data. The presence of both marks at the promoter region, representing a poised state, is shown in control cells for *Fev, Chga, Chgb,* and *Abat* genes associated with endocrine progenitors. In contrast, a poised state is shown at promoter regions of genes *Pyy* (α cells); *Celsr1,* and *Glp1r* (markers of ß cells) in *Isl1CKO* but not control cells. A silencing H3K27me3 mark is located at the promoter region of a key ß-cell maturation marker *Ucn3* only in *Isl1CKO* cells

Next, we analyzed the transcriptome of pancreatic endocrine cells at P9, when a mature functional glucose-stimulated-insulin-secretion phenotype of β cells is acquired [35]. We identified 1049 protein-coding genes differentially expressed in endocrine cells, comparing *Isl1CKO* to controls (Fig. 7a; Additional file 6: Dataset S1d). GO analysis of the differentially downregulated genes showed a sustained downregulation of gene classes involved in insulin secretion, peptide secretion, and transport, including reduced levels of all major hormone transcripts *Gcg, Ins1, Ins2, Ppy*, and *Sst* (Fig. 7b, c; Additional file 6: Dataset S1e). The deficiency in endocrine hormone production corresponds with observed abnormalities in the islets of Langerhans and severe postnatal diabetic phenotype of *Isl1CKO* (Fig. 2). Although differentiated insulin-producing ß cells were found in *Isl1CKO*, we next wanted to elucidate the maturation state of these cells. Some of the transcription factors that have been reported to regulate mature ß-cell function, such as *Mafa, Pparg, Bcl6, Pdx1* [38], together with mature ß-cell markers *Ucn3, Slc2a2, Trpm5,* and *G6pc2* [35, 47] were downregulated in *Isl1CKO* (Fig. 7c, Additional file 6: Dataset S1d), suggesting abnormalities in the transition between immature and mature ß cells. PDX1 is critical for inducing and maintaining ß-cell maturation and identity by regulating target genes, such as *Slc2a2*, *Mafa*, and *Ins1* [48, 49]*.* The immature state of the ß-cell population in *Isl1CKO* was further indicated by the increase of *Wif1, Dkk3, Tgfbr3*, and *Tab3* associated with Wnt and TGF-ß signaling pathways that are normally downregulated in mature ß cells [38]. Furthermore, the most enriched GO categories for the upregulated transcripts in *Isl1CKO* endocrine cells were involved in biological processes related to development (Fig. 7b, Additional file 6: Dataset S1e). Consistent with an immature transcriptomic signature, *Isl1CKO* endocrine cells displayed an enrichment for genes defining intermediate endocrine progenitors, such as *Fev, Tle1, Evpl, Sez6l, Gc,* and *Vim* (Fig. 7d, Additional file 6: Dataset S1f), suggesting abnormalities in the progression of endocrine cell differentiation.

To determine whether the absence of *Isl1* had an impact on the chromatin architecture of postnatal endocrine cells, we performed CUT&Tag-seq of H3K4me3 and H3K27me3 marks in FACS-sorted tdTomato^+^ cells from the P9 pancreas (Additional file 1: Fig. S5). To correlate changes in gene expression with changes in H3K4me3 and H3K27me3 patterns in *Isl1CKO* endocrine cells, we focused on H3 methylation states in the promoter regions of differentially expressed genes at P9 (Fig. 7e). 62% of differentially expressed genes were found to have either or both H3K4me3 and H3K27me3 marks at their promoter regions, and 64% of those genes acquired distinct H3 modification patterns between control and *Isl1CKO* (Fig. 7e, f). Next, we correlated distinct H3K4me3 and H3K27me3 modifications at the promoter regions with changes in expression of signature genes associated with endocrine progenitors, α and ß cells (Fig. 7g). We identified transcription activator H3K4me3 marks at the promoter regions of endocrine progenitor genes, which had upregulated expression in *Isl1CKO* cells in contrast to these genes in endocrine cells from control mice which exhibited a suppressive histone methylation pattern associated with either loss of H3K4me3 or acquisition of H3K27me3 marks. Notably, the promoter of the signature gene *Fev,* characterizing the endocrine progenitor lineage [5, 36, 37], was transcriptionally poised in endocrine cells from the control pancreas by acquiring a suppressive methylation mark H3K27me3 (Fig. 7g, h). Consistently, a silencing methylation signature of H3K27me3 was acquired by control endocrine cells at promoter regions of *Chgb* and *Chga.* Although *Chga* and *Chgb* are often utilized as markers of differentiated endocrine lineages, these genes were highly expressed in *Isl1CKO* endocrine cells compared to controls, presumably reflecting an immature endocrine lineage expression profile [36]. In correlation with epigenetic repression by H3K27me3, these genes exhibited diminished expression in control cells compared to *Isl1CKO*. Thus, these observations suggested that control cells are becoming terminally differentiated compared to those of *Isl1CKO*. In *Isl1CKOs*, we found a bivalent state at promoter regions of several downregulated ß- and α-cell genes, including *Celsr1*, *Glp1r*, and *Pyy* (Fig. 7g, h). Interestingly, a silencing methylation signature of H3K27me3 was uniquely acquired at the promoter region of a key ß-cell maturation marker *Ucn3* in *Isl1CKO* cells, indicating the contribution of epigenetic repression to the regulation of ß-cell maturation.

### ISL1 directly targets regulatory elements of critical genes involved in endocrine development

**Fig. 8:**
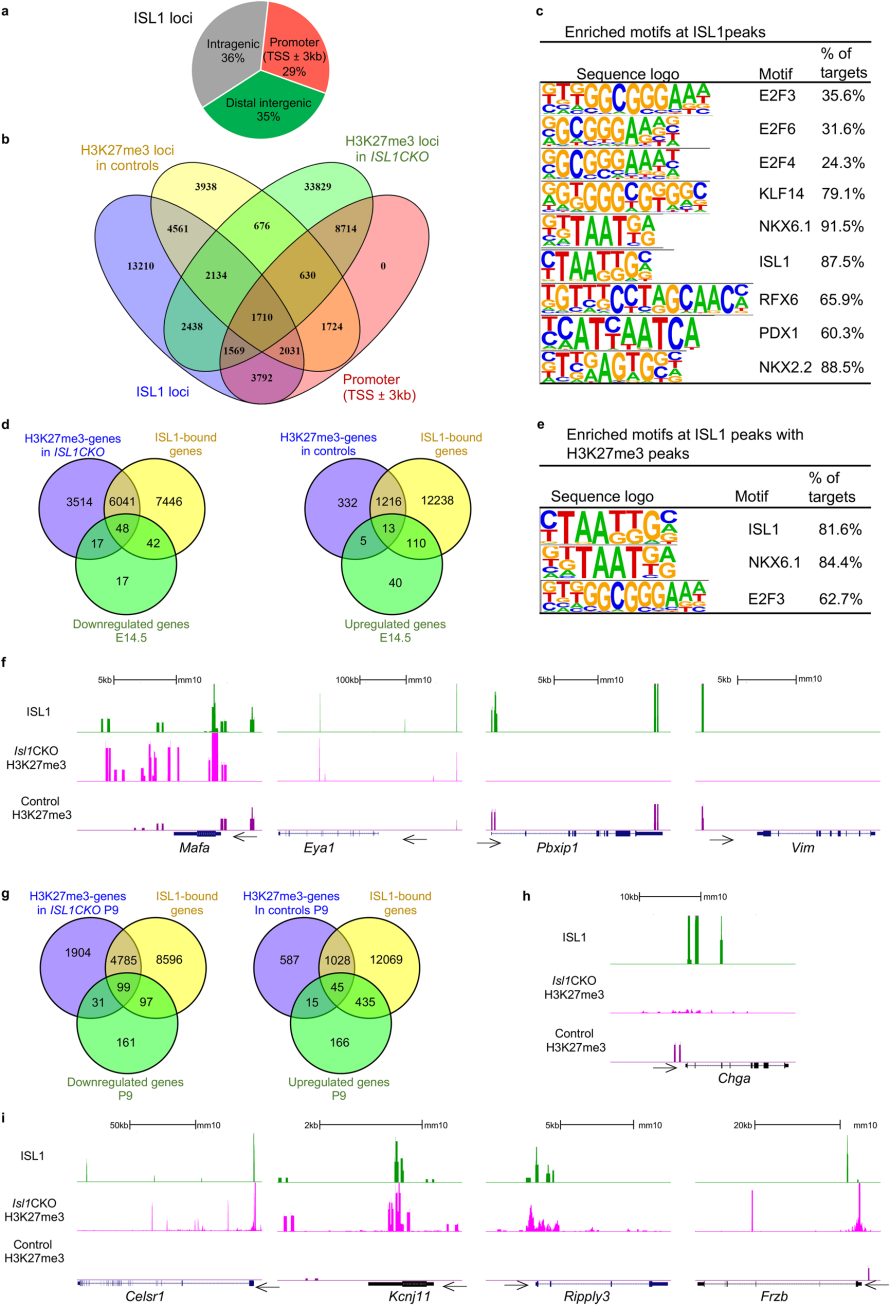
ISL1 binds regulatory elements of critical developmental genes and reprograms the H3K27me3 landscape in endocrine cells. **a** Pie chart showing genomic distribution of ISL1 loci. **b** Venn diagram indicating overlap of ISL1-loci, and of distinctive H3K27me3 loci from pairwise comparisons control and *Isl1CKO* endocrine cells at E14.5 in promoter regions (Additional file 7: Dataset S2). **c** Sequence logos of the significantly enriched motifs against ISL1 peaks (2 kb areas) from Homer FindMotifsGenome analysis. Percent of target sites in ISL1 peaks is indicated. **d** Venn diagrams illustrating intersection of ISL1-bound genes, genes with differential H3K27me3 depositions from pairwise comparisons of control and *Isl1CKO* endocrine cells at E14.5, and differentially expressed genes at E14.5 identified from RNA-seq data. **e** Sequence logos of the significantly enriched motifs against ISL1 peaks containing H3K27me3 peaks (2 kb areas) from Homer FindMotifsGenome analysis. **f** Genome track view of representative gene loci showing ISL1 and H3K27me3 normalized read peaks based on CUT&Tag-seq data from E14.5 endocrine cells (an arrow indicates a TSS). The presence of both marks at the promoter region indicates overlapping occupancy of silencing methylation signature of H3K27me3 and ISL1. **g** Venn diagrams indicating overlap of ISL1-bound genes and genes with distinctive H3K27me3 depositions from pairwise comparisons P9 control and *Isl1CKO* endocrine cells, and differentially expressed genes identified from RNA-seq data at P9 (Additional file 8: Dataset S3). **h** and **i** Genome track view of representative gene loci showing ISL1 and H3K27me3 normalized read peaks based on CUT&Tag-seq data. The presence of both marks at the promoter region indicates overlapping occupancy of silencing methylation signature of H3K27me3 and ISL1 in upregulated (h) and downregulated (i) genes. Samples used for comparison: ISL1-CUT&Tag-seq E14.5 endocrine cells, and H3K27me3-CUT&Tag-seq P9 endocrine cells

To understand the molecular modes of action of ISL1 during endocrine development, we then performed ISL1 CUT&Tag-seq to map the genome-wide binding of ISL1 in FACS-sorted tdTomato^+^ cells of the E14.5 pancreas (Additional file 7: Dataset S2). In peak calling, 31,445 ISL1-occupied loci were identified. ISL1 binding sites were found at both promoter and non-promoter regions, with approximately 29% of ISL1-loci were annotated within 3 kb from the transcription start site (TSS) of a gene, 36% of ISL1-loci detected in introns and exons, and 35% in distal intergenic regions (Fig. 8a, Additional file 1: Fig. S6a). Comparing the distribution of H3K27me3 loci and ISL1 loci at gene promoters between control and *Isl1CKO* mutant, we found that approximately 6.5% (2031 loci) and 5% (1569 loci) of ISL1-binding sites were associated with differential H3K27me3 depositions at promoter regions between control and *Isl1CKO*, respectively (Fig. 8b). Identified ISL1-loci were annotated to 13,577 genes. Many genes with ISL1 bound at their promoter regions represent critical regulators in development, such as transcription factors, signaling molecules, epigenetic modifiers, and members of the SWI/SNF chromatin remodeling complex. These ISL1 targets included key regulators in pancreatic endocrine development, such as *Pax6, Mafb, Nkx6.2, Insm1, Sox9, Arx, Fev, Nkx6.1, Foxa2, Neurod1, Rfx3,* and *Rfx6* [1]. Among the most enriched transcription factor motifs at the sites occupied by ISL1 were consensus binding sites for the E2F family, KLF14, and NKX6.1 (Fig. 8c). For example, NKX6.1 is essential for both early and late stages of pancreatic development with a critical role in the formation of β cells [50–52], KLF14 is an important regulator of metabolic diseases, such as diabetes and obesity[53, 54], and E2F3 activates β-cell proliferation [55]. High percentage of ISL1-bound regions were enriched for transcription factor PDX1 motif, β-cell-fate determining transcription factor [49], and NKX2.2 and RFX6 essential for endocrine cell development and β-cell-fate [56–58]. To investigate ISL1 activity further, we compared genes with differential histone H3K27me3 patterns between control and *Isl1CKO*, genes with ISL1 binding sites, and differentially expressed genes identified from RNA-seq (Fig. 8d). 73% genes deregulated in *Isl1CKO* endocrine cells were bound by ISL1. 53% downregulated genes were associated with *Isl1CKO*-distinctive H3K27me3 marks and were bound by ISL1, indicating an intriguing association between *Isl1* deficiency and H3K27me3 repressive epigenetic state. The most enriched GO Biological processes for ISL1-bound genes with *Isl1CKO*-distinctive H3K27me3 depositions were related to cell fate determination, embryonal development, and morphogenesis (Additional file 1: Fig. S6b). In contrast, only 13% of ISL1-bound upregulated genes (14 genes) exhibited distinctive H3K27me3 modifications. NKX6.1, a homeobox-containing transcription factor, was found to be one of the most enriched motifs with a high percentage of ISL1 peaks and H3K27me3 depositions (Fig. 8e). NKX6.1, the ISL1 target gene, plays a crucial role in regulating the chronological development of different endocrine cell types [50], and initiates and maintains β cell-specific gene expression programs while repressing programs of alternative endocrine lineages [52]. These data indicate that ISL1 and NKX6.1 may interact during pancreas endocrine cell development. *Isl1CKO*-distinctive H3K27me3 signatures were found in 48 downregulated ISL1 target genes, which are key regulators of endocrine development, including *Mafa, Ripply3, Sorcs1, Phox2b, Eya1*, and *Mnx1* (Fig. 8f). In contrast, distinctive silencing H3K27me3 marks observed in control endocrine cell samples were detected in 13 upregulated ISL1 targets, including *Vim* and an inhibitor of the PBX1 homeodomain transcription factor, *Pbxip1* [59] (Fig. 8f). It is worth noting that some endocrine progenitor signature genes that were upregulated in *ISL1CKO* contained ISL1 binding sites at their promoter regions, among them *Fev*, *Apoe*, *Chgb*, *Chga, B2m*, *Gprc5a*, *Wif1*, and *Ttr* (genes are marked by asterisks in Fig. 6e; Additional file 7: Dataset S2). These data indicate that *Isl1* deficiency was associated with the remodeling of epigenetic H3K27me3 marks and altered gene expression levels of ISL1 target genes, contributing to the dysregulation of endocrine development in *Isl1CKO* mice.

Next, we wanted to investigate the extent of shared ISL1-bound genes between embryonic E14.5 endocrine cells and functionally matured P9 endocrine cells. To accomplish this, we compared the set of ISL1-bound genes with genes that displayed differential H3K27me3 patterns between P9 control and *Isl1CKO*, as well as differentially expressed genes identified from RNA-seq data in P9 endocrine cells (Fig. 8g, Additional file 8: Dataset S3). The intersection of the RNAseq data with CUT&Tag data revealed that 73% (480 genes) of upregulated and 51% (196 genes) of downregulated genes were bound by ISL1, respectively. Downregulated genes with ISL1 bound at their promoter regions included genes associated with insulin secretion and β-cell function (Fig. 7b), such as *Abcc8*, *Acvr1c*, *Cdk16*, *Kcnj11*, *Nnat,* and *Mafa* [60, 61]. ISL1-bound promoters of upregulated genes associated with endocrine progenitor state included *Fev, Chga, Chgb, Cldn4, Pbxip1, Gpc4, Tle1*, and *Wif1* (Additional file 8: Dataset S3). 25% (99 genes) of downregulated genes had ISL1 binding sites and contained *Isl1CKO*-specific-H3K27me3 silencing marks, while 7% (45 genes) upregulated genes had ISL1 binding sites and contained control-specific-H3K27me3 silencing marks (Fig. 8g–i). This analysis revealed a significant enrichment of H3K27me3 silencing marks in ISL1-bound genes that were downregulated in P9 *Isl1CKO* endocrine cells, highlighting the critical role of ISL1 in regulating functional glucose-stimulated-insulin-secretion phenotype of β cells and the diabetic phenotype of *Isl1CKO*.

## Discussion

**Fig. 9:**
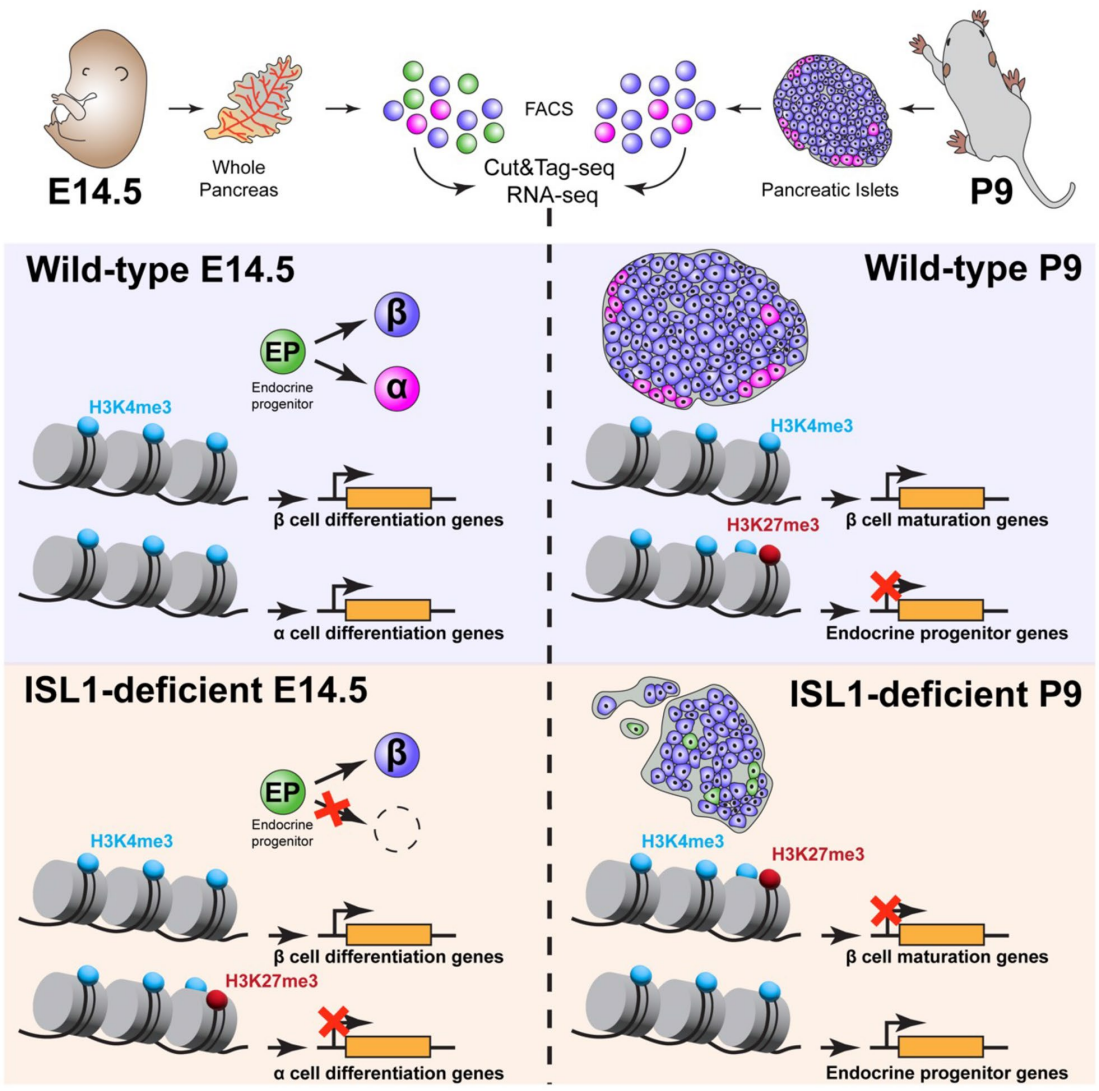
Schematics of changes induced by the elimination of *Isl1* in pancreas development

Cell replacement or in vivo differentiation of cells in the islets of Langerhans necessitate understanding of molecular programs driving pancreatic endocrine differentiation and maturation. Although signaling and transcription factor networks regulating different stages of pancreatic development and differentiation of islet cell types have been well studied, the unique role of transcription factors in programming the epigenome during lineage differentiation is largely unexplored. This study sought to determine how ISL1 regulatory networks control α- and ß-cell differentiation and how *Isl1*-deficiency cause endocrine progenitors to fail to produce functional α- and ß-cells, resulting in the severe diabetic phenotype associated with *Isl1* deletion [31]. Here we show that ISL1 drives α cell differentiation and controls the acquisition of the β-cell mature endocrine phenotype. Using RNA sequencing together with CUT&Tag DNA sequencing, we uncovered changes in the epigenetic landscape of H3K4me3 and H3K27me3 modifications at promoter regions correlating with differential gene expression in *Isl1CKO*. Additionally, we explored multifaceted roles of ISL1 in epigenetic and transcriptional regulations by genome-wide profiling of ISL1 binding. These results indicated that the absence of *Isl1* resulted in changes in transcriptional networks and epigenetic remodeling that may contribute to the regulation of gene expression and abnormalities in endocrine cell differentiation in the pancreas of *Isl1CKO* (see graphic summary in Fig. 9).

There are two main contributions of our research to the understanding of ISL1 function. First, we are first to provide the evidence that ISL1 regulates an advancement towards the late stage of α-cell differentiation. Second, we are the first to report that ISL1 controls the maturation of β-cells. In terms of our first main contribution, transcriptome analyses of *Isl1CKO* endocrine cells at E14.5 revealed a shift towards the endocrine progenitor population at the expense of α-cell differentiation during the secondary transition of pancreas development of *Isl1CKO*. We found increased levels of *Fev* in *Isl1CKO* endocrine cells, associated with a transitory intermediate endocrine progenitor population before branching into α and ß differentiated endocrine cell types [25, 36, 37, 39]. For example, FEV^+^ endocrine progenitors co-expressing *Peg10* tend to differentiate into α cells [36]. Our lineage reconstruction clustering analysis showed that α cell development was arrested in the early stage of α-cell differentiation, as we did not find any late α cells associated with high expression of *Mafb*, *Meis2*, and *Scgn* [37, 39] and expression of α-cell fate promoting transcription factors *Peg10* and *Pou6f2* [36, 40] was reduced in *Isl1CKO* endocrine cells. Motif enrichment analyses identified ISL1 and FEV as the top-ranking transcription factors associated with differentially expressed genes in *Isl1CKOs* at E14.5, suggesting possible functional interactions of ISL1 and FEV. *Fev* is an ISL1 direct target gene, as shown by our ISL1 CUT&Tag-seq. We uncovered that ISL1 directly binds promoters of key regulatory genes for pancreatic endocrine development, including *Pax6, Mafb, Nkx6.2, Insm1, Sox9, Arx, Fev, Nkx6.1, Foxa2, Neurod1, Rfx3,* and *Rfx6* [1, 3, 5]*.* Additionally, motif analyses revealed the enrichment of motifs for NKX6.1 [50, 52], RFX6 [42, 56, 58], PDX1 [10, 49], and NKX2.2 [14, 57], critical regulators of endocrine cell development, at the sites occupied by ISL1, suggesting their cooperation with ISL1. Downregulated genes in *Isl1CKO* endocrine cells had enhanced depositions of silencing H3K27me3 in ISL1-bound genes. This suggests that the absence of ISL1 can alter the epigenetic landscape of endocrine cells during differentiation. For example, the ISL1 binding sites and H3K27me3 depositions were associated with downregulated genes defining the α- and β-cell lineages, among them *Mafa, Meis2, Ripply3*[62]*, Eya1, Phox2b*, and *Mnx1*. Distinct regulatory patterns were observed in the upregulated genes associated with intermediate states of endocrine differentiation, including *Chgb, Chga, Vim, Pbxip1*, and *Fev*. Specifically, these genes exhibited ISL1 binding sites and a repressive H3K27me3 mark at their promoter regions in control endocrine cells. Our results further suggested that changes in distinct histone H3K4me3 and H3K27me3 methylation patterns at promoter regions of transcriptional regulators, such as *Mafa, Ripply3, Phox2b*, and *Mnx1* contributed to regulation of gene expression in *Isl1CKO*. Thus, we have shown that deletion of *Isl1* at the onset of endocrine cell formation results in a loss of multiple markers of α-cell identity characterized by the absence of glucagon expressing cells, and in a shift in the transcriptomic signature towards intermediate FEV^+^ progenitor states.

In terms of our second main contribution, ISL1 controls acquisition of the β-cell mature endocrine phenotype. At P9, the characteristic core-mantle organization of the islets with the β-cell core and α cells mainly at the islet periphery was disrupted in *Isl1CKO* together with reduced insulin and PDX1 levels in β cells. Our transcriptome profiling of *Isl1CKO* endocrine cells at P9 demonstrated compromised regulation of insulin secretion, including downregulation of transcription factors *Pdx1* and *Mafa*. Both *Pdx1* and *Mafa* are direct transcriptional targets of ISL1 in adult β cells [31, 62]. Although *Mafa* has been suggested as a possible factor contributing to the development of compromised β cells [31], it cannot explain neonatal diabetic phenotype of *Isl1*-deficient mice. Glucose-regulated insulin secretion, β-cell mass, and islet cell architecture are compromised only in adult mice with pancreas-specific *Mafa* deletion [60], in contrast to the neonatal diabetic phenotype in a β-cell-specific *Pdx1* deletion mutant [63]. Notably, pancreas-specific knockout *Mafa* mice are glucose intolerant but have normal fasting glucose levels [60], whereas loss of PDX1 from pancreatic β cells causes overt hyperglycemia in different models [48, 49, 63]. Based on our results, we postulate that ISL1 regulates PDX1 expression during pancreatic β-cell maturation. PDX1 is critical to induce and maintain ß-cell maturation and identity [48, 49]*.* Compared to endocrine cells from the control pancreas, expression of ß-cell maturation markers, such as *Ucn3, Trpm5,* and *G6pc2* [35, 47], was reduced in *Isl1CKO,* suggesting that ISL1 plays an important role in the maturation of ß cells. Additionally, we found an enrichment of endocrine progenitor signature genes, such as *Fev, Chga/b, Vim, Cldn4,* and *Sez6l* in *Isl1CKO* cells, representing an intermediate transition state endocrine progenitor population [25, 36, 37]. This transcriptional profile indicated that *Isl1CKO* cells might be “trapped” in a transition endocrine progenitor state. Consistent with the immature endocrine transcriptomic signature, loss of *Isl1* was associated with significantly reduced levels of all major hormone transcripts *Gcg, Ins1, Ins2, Ppy*, and *Sst.* In addition, a silencing H3K27me3 modification was uniquely acquired at the promoter region of *Ucn3* in *Isl1CKO* cells. Consistent with the idea that pancreatic endocrine cells display a mature functional glucose-stimulated-insulin-secretion phenotype at P9 [35], signature genes of endocrine progenitors, such as *Fev, Chga*, and *Chgb* [36], which have ISL1 binding sites at their promoter regions, were marked by a suppressive H3K27me3 mark in endocrine cells from the control P9 pancreas in contrast to an active state (H3K27me3^−^ H3K4me3^+^) in *Isl1CKO* cells.

The limitation of this study is that bulk-cell sequencing approaches provide an average of molecular differences from multiple cells. Although we applied the deconvolution method to our bulk-cell RNA-seq data to estimate the cell proportion and transcriptomic signatures of major cell types in the developing pancreas, future single-cell RNA-seq analyses are needed to fully establish molecular differences linked to specific cell states, cell-to-cell variability, and uncover the pathways of cell lineage differentiation affected by *Isl1* deletion.

## Conclusions

Significantly advancing previous research [31], our study has revealed the molecular bases of two different regulatory roles of ISL1 during the development of pancreatic endocrine cells. First, ISL1 controls an α-cell lineage fate. Our lineage reconstruction clustering analysis of *Isl1CKO* endocrine cells showed that α cell development was arrested in the early stage of α-cell differentiation, which was associated with a loss of multiple markers of α-cell identity that was characterized, in turn, by the absence of glucagon expressing cells during the secondary transition at E14.5. Second, ISL1 regulates the acquisition of the β-cell mature phenotype. At P9, when a mature functional glucose-stimulated-insulin-secretion phenotype of β cells is acquired [35], transcriptome profiling of *Isl1CKO* endocrine cells identified downregulation of key ß-cell regulators and mature ß-cell markers. Most importantly this was particularly the case of *Pdx1,* which is necessary for establishing and maintaining mature β cells [49, 63]*.* Additionally*,* an enrichment of endocrine progenitor signature genes towards FEV^+^ intermediate progenitor states indicates a shift in the transcriptomic signature in *Isl1CKO*. Such findings suggest abnormalities in the transition between immature and mature ß cells and correlate with the progressive decline of ß-cell function and diabetic phenotype of *Isl1CKO*. Moreover, our study provides the first insights into the function of ISL1 directly or indirectly orchestrating chromatin remodeling in correlation with gene expression changes during pancreatic endocrine development. Altogether, these results represent compelling evidence that ISL1 transcriptionally and epigenetically controls pancreas endocrine development, affecting α-cell lineage fate decisions and maturation processes of ß cells. Future studies are needed to determine how ISL1 modulates the epigenetic landscape and how *Isl1* deficiency affects transcriptomic signatures of major cell types in the developing pancreas.

## Methods

### Experimental model

Animal experiments were conducted according to protocols approved by the Animal Care and Use Committee of the Institute of Molecular Genetics, Czech Academy of Sciences. All experiments were performed with littermates (males and females) cross-bred from two transgenic mouse lines: floxed *Isl1* [*Isl1*^*f/f*^; *Isl1*^*tm2Sev*^, Stock No: 028501 Jackson Laboratory, [15]], and Neurod1-Cre [Tg(Neurod1-cre)1Able, Stock No: 028364 Jackson Laboratory, [32]]. Lines were maintained on C57BL/6 background. Neurod1-Cre mice do not have any detectable phenotype. Breeding scheme: Female mice *Isl1*^*f/f*^ were crossed with *Isl1*^*f/*+^; *Neurod1-Cre* males, in which, *Neurod1-cre* knock-in allele was inherited paternally to minimize the potential influence of maternal genotype on the developing embryos. *Isl1*^*f/*+^ or *Isl1*^*f/f*^ mice were used as the controls. The reporter tdTomato line (Ai14, B6.Cg-*Gt(ROSA)26Sor*^*tm14(CAG−tdTomato)Hze*^, Stock No: 7914 Jackson Laboratory) was used. Genotyping was performed by PCR on tail DNA (Additional file 1: Table S1). Mice were kept under standard experimental conditions with a constant temperature (23–24 °C) and fed on soy-free feed (LASvendi, Germany). The females were housed individually during the gestation period and the litter size was recorded. Blood glucose levels were measured in animals by glucometer (COUNTOUR TS, Bayer); blood glucose levels maintained above 13.9 mmol/L are classified as diabetic. For total pancreatic insulin content, pancreases were excised, weighed, minced, and homogenized in acid–ethanol. A hormone concentration in extracts was measured by ELISA using Mouse Insulin ELISA kit (Mercodia, Sweden).

### Reverse transcription-quantitative Real-Time Polymerase Chain Reaction

RT-qPCR was performed as described previously [64]. Briefly, total RNA was isolated from the whole pancreas at E12.5 and E14.5 or (n = 8 samples/group) by Trizol RNA extraction. Following RT, quantitative real-time PCR (qPCR) was performed with the initial AmpliTaq activation at 95 °C for 10 min, followed by 40 cycles at 95 °C for 15 s and 60 °C for 30 s, as described. The *Hprt1* gene was selected as the best reference gene for our analyses from a panel of 12 control genes (TATAA Biocenter AB, Sweden). The relative expression of a target gene was calculated based on qPCR efficiencies and the quantification cycle (Cq) difference (Δ) of an experimental sample versus a control. Primers were designed using Primer Blast tool (https://www.ncbi.nlm.nih.gov/tools/primer-blast/). Primers were selected according to the following parameters: length between 18 and 24 bases, melting temperature (Tm) between 58° and 60 °C, G + C content between 40 and 60% (optimal 50%) and efficiency above 80%. Primer sequences are presented in Additional file 1: Table S2.

### Immunohistochemistry and morphological evaluations

For vibratome sections, dissected tissues were fixed in 4% PFA, embedded in 4% agarose gel and sectioned at 80 µm on a Leica VT1000S vibratome. All company names and catalog numbers of primary and secondary antibodies, and their dilutions used in this study, are in Additional file 1: Table S3. The nuclei were counterstained with Hoechst 33342. Image acquisition was completed using the Zeiss LSM 880 NLO scanning confocal microscope, with ZEN lite software. The number of glucagon (GCG) and insulin (INS) expressing cells, and Ki67^+^ cells were counted in one vibratome section of *Isl1CKO* and control embryos or mice (n = 5 pancreases per genotype and for each age) with the largest pancreatic footprint per individual using the Cell Counter plugin of Image J (NIH). The number of INS and pHH3 expressing cells were counted in vibratome sections of *Isl1CKO* and control pancreases at E17.5 (n = 3 pancreases per genotype) and P0 (n = 4 pancreases per genotype). The number of NEUROD1^+^/ISL1^+^ cells at E10.5 were counted in the whole mount of the dorsal pancreas (n = 5 pancreases per genotype) using the Cell Counter plugin of Image J (NIH). For the evaluation of glucagon delaminating cells at E11.5 were quantified using the thresholding tool Image J (NIH) and expressed as a percentage of the total GCG^+^ area to PDX1^+^ area (n = 9 control pancreases; n = 8 *Isl1CKO* pancreases).

### Light-sheet fluorescent microscopy (LFSM) and analysis of images

The pancreas was microdissected from control-*Ai14* and *Isl1CKO-Ai14* mice (postnatal day P0). We used an advanced CUBIC protocol [65] for tissue clearing to enable efficient imaging by light-sheet microscopy. Briefly, the microdissected tissue was fixed in 4% PFA for 1 h, washed with PBS, and incubated in a clearing solution Cubic 1 for 5 days at 37 ℃. Before immunolabeling, samples were washed in PBT (0.5% Triton-X in PBS) 4 × for 30 min. In addition to *tdTomato* expression, cleared samples were immunolabeled using different combinations of antibodies (anti-INS, anti-GLP1, and anti-TUBB3). Samples were stored before imaging in Cubic 2 at room temperature. Zeiss Lightsheet Z.1 microscope with illumination objective Lightsheet Z.1 5x/0.1 and detection objective Dry objective Lightsheet Z.1 5x/0.16 was used for imaging at the Light Microscopy Core Facility of the Institute of Molecular Genetics of the Czech Academy of Sciences. IMARIS software v8.1.1 (Bitplane AG, CA, USA) was used for image processing.

### Isolation of pancreatic endocrine cells

The pancreases from P9-P10 pups were first perfused by 1 mg/ml collagenase in Hank’s balanced salt solution, dissected, and incubated at 37 °C for 10–12 min to release endocrine cells/islets. Digested pancreatic tissue was washed 3 × by 1% FBS in Hank’s solution. To generate single cells, the tissue was further dissociated by trypsinization as described [66]. Briefly, tissue was dissociated using 0.05% trypsin/0.53 mM EDTA at 37 °C for 5 min. Digestion was stopped by the FACS buffer (2% FBS and 10 mM EGTA in PBS [66]), and cells were then 1 × washed by FACS buffer. The pancreases microdissected from E14.5 embryos were directly trypsinized and prepared for FACS as described above. Finally, cell suspensions were filtered through 40 µm nylon mesh and immediately tdTomato^+^ cells were sorted using a flow cytometer (BD FACSAria™ Fusion), through a 100 µm nozzle in 20 psi, operated with BD FACSDiva™ Software (Additional file 1: Fig. S7). For RNA sequencing, 100 sorted cells were collected into individual wells of 96-well plate containing 5 µl of lysis buffer of NEB Next single-cell low input RNA library prep kit for Illumina (New England Biolabs #E6420). Plates were frozen immediately on dry ice and stored at − 80 °C. The total time from euthanasia to cell collection was ∼3 h. For the epigenetic study, on average, 4700 cells/sample at E14.5 and 14,700 cells/sample at P9 were sorted. Cell sorting was performed in the Imaging Methods Core Facility at BIOCEV.

### RNA sequencing and analyses

RNA-seq libraries were prepared from 100 FACS-sorted cells/sample obtained from the pancreases of reporter *Isl1CKO-Ai14* mutant (n = 5 samples) and reporter control-*Ai14* (n = 6) from E14.5 embryos; and *Isl1CKO-Ai14* mutant (n = 6) and reporter control-*Ai14* (n = 5) from P9 mice. Each sample contained 100 tdTomato^+^ endocrine cells. Following the manufacturer's instructions, the NEB Next single-cell low input RNA library prep kit for Illumina was used for cDNA synthesis, amplification, and library generation [67] at the Gene Core Facility (Institute of Biotechnology CAS, Czechia). Fragment Analyzer assessed the quality of cDNA libraries. The libraries were sequenced on an Illumina NextSeq 500 next-generation sequencer. NextSeq 500/550 High Output kit 75 cycles (Illumina #200,024,906) were processed at the Genomics and Bioinformatics Core Facility (Institute of Molecular Genetics CAS, Czechia). RNA-Seq reads in FASTQ files were mapped to the mouse genome using STAR [version 2.7.0c [68]] GRCm38 primary assembly and annotation version M8. The raw data of RNA sequencing were processed with a standard pipeline. Using cutadapt v1.18 [69], the number of reads (minimum, 32 million; maximum, 73 million) was trimmed by Illumina sequencing adaptor and of bases with reading quality lower than 20, subsequently reads shorter than 20 bp were filtered out TrimmomaticPE version 0.36 [70].

Ribosomal RNA and reads mapping to UniVec database were filtered out using bowtie v1.2.2. with parameters -S -n 1 and SortMeRNA [71]. A count table was generated by Rsubread v2.0.1 package using default parameters without counting multi mapping reads. The raw RNA-seq data were deposited at GEO: (https://www.ncbi.nlm.nih.gov/geo/).

DESeq2 [v1.26.0 [72]] default parameters were used to normalize data and compare the different groups. Genes were then filtered using the criteria of an adjusted P-value P_adj_ < 0.05, and a base mean ≥ 50, and Fold change > 1.5 for upregulated genes and < 0.5 for downregulated genes for both E14.5 and P9 data to identify differentially expressed genes between *Isl1CKO* and control endocrine cells. The enrichment of the functional categories and functional annotation clustering of the differentially expressed genes was performed using g: Profiler [73] using version e104_eg51_p15_3922dba with g: SCS multiple testing correction methods applying a significance threshold of 0.05. Transcription factor (TF) enrichment analysis (TFEA) [41] was used to identify the enrichment of TF target genes in our set of differentially expressed genes. The top seven enriched TFs are listed (Additional file 6: Dataset S1c).

### Deconvolution of endocrine cell subtypes

Deconvolution was performed using the CibersortX algorithm at cibersortx.stanford.edu [74]. Single-cell transcriptomic profiling dataset of cells in the embryonic pancreas [39] was used as a reference, including count matrix and metadata labels. Particularly, only cells with pancreatic epithelial or endocrine cell fate were used, corresponding to the annotation of five broader cell types—α cells, β cells, endocrine progenitors, trunk epithelium and tip epithelium [39]. The reference matrix was built out of the 2589 cells and gene list of 18,565 gene features, as deposited by [39]. Each cell population counted > 250 cells. The units of the reference matrix were UMI counts. Calculation of the scRNA-seq signature matrix was done in default mode (quantile normalization disabled, minimal expression of 0.75, replicates of 5, sampling of 0.5). Imputation of cell fractions and group-mode expression were used in default settings, with S-mode batch correction enabled, quantile normalization disabled and n = 100 permutations for significance analysis. Sample mixture file was submitted with unfiltered gene list 27,124 features for *Isl1CKO* and in UMI counts.

### Cut&Tag sequencing and analyses

Bench top CUT&Tag version 3 was performed as previously described [46, 75], with minor modifications. Specifically, nuclei from freshly FACS-sorted tdTomato^+^ pancreatic endocrine cells were captured by Concanavalin A-coated magnetic beads to facilitate subsequent washing steps and the reaction was carried out in 0.2 ml PCR tubes. CUT&Tag validated primary antibodies anti-H3K4me3 (Active Motif, #39,159, 1:100), Anti-H3K27me3 (Active Motif, #39155, 1:100), anti-ISL1 (Developmental Hybridoma Bank, #39.4D5, 1:50), normal rabbit IgG negative control (EpiCypher, #13–0042, 0.5 µg/reaction), anti-mouse secondary antibody (EpiCypher, #13–0048, 0.5 µg/reaction), and anti-rabbit secondary antibody (EpiCypher, #13-0047, 0.5 µg/reaction). Binding of pAG-Tn5 (EpiCypher, 15-1017, 2.5µL/reaction) was at RT for 60 min followed by tagmentation. To stop tagmentation and solubilize DNA fragments, 1.67 μL 0.5 M EDTA, 0.5 μL 10% SDS and 0.42 μL 20 mg/mL Proteinase K (20 mg/mL) was added to each sample. Samples were incubated for 1 h at 55 ºC to digest (and reverse cross-links), followed by DNA precipitation and purification. DNA was dissolved in 22 μL of 1 mM Tris–HCl pH 8, 0.1 mM EDTA buffer and utilized as template for library generation with Universal i5 Primer and Uniquely Barcoded i7 Primers for Illumina. DNA libraries were sequenced on the MiSeq, Illumina using MiSeq Reagent Kit v3, which allows extend read lengths up to 2 × 75 bp at the OMICS Genomics facility (BIOCEV). CUT&Tag-seq H3K27me3 data are from two independent biological replicates and CUT&Tag-seq H3K4me3 data are from one biological sample, each sample was pooled together from five to nine pancreases of E14.5 embryos (〜 4700 cells/sample) and from two to three pancreases of P9 mice per genotype (〜 14,700 cells/sample). CUT&Tag-seq ISL1 data are from two independent biological replicates, each biological sample was pooled together from eleven E14.5 pancreases (〜 6100 cells/sample).

Data analyses were performed following CUT&Tag Data processing tutorial [76]. Paired-end sequencing data were mapped using bowtie2 (version 2.2.5) [77] to mouse genome GRCm38 primary assembly. PCR duplicates were not removed. After filtering and conversion to bedgraph format, peak calling was performed with usage of relevant IgG controls and stringent mode with tool SEACR (version 1.3) [78]. Peaks were annotated using CHIPseeker (version 1.30.3) [79] and annotation version M8 of mouse genome. Enriched peak detection was performed using EdgeR (version 3.36.0) [80] with filtering criteria for H3K4me3 modification > 25 CPM (count per million). Enrichments in H3K27me3 peaks between control and *Isl1CKO* were identified in the pairwise comparisons as a peak difference equal to or greater than threefold. Comparative analyses of ISL1 and H3K27me CUT&Tag-seq data were done as follows. After filtering of ISL1 CUT&Tag data and conversion to bam format, bam files from replicates were merged with samtools (version 1.6). Peak calling was performed with usage of relevant IgG controls and applying the –keep-dup all –nomodel –extsize 200 settings to the callpeak command in MACS2 (version 1.3) [78]. Peaks were annotated using CHIPseeker (version 1.30.3) [79] and annotation version M8 of mouse genome. Normalization of samples was performed using EdgeR (version 3.36.0) [80]. Sum of normalized counts of all peaks in a gene was performed for detecting epigenetic enrichment at the gene level. A gene was considered as enriched for H3K27me3 between control and *Isl1CKO* when the total levels was equal to or greater than fivefold. Motif enrichment analysis was performed using the findMotifsGenome.pl function in HOMER (version 4.11). Bed files with ISL1 peak summits were used as input to look for motif enrichment in a 2 kb area.

### Experimental design and statistical analyses

All comparisons were made between animals with the same genetic background, typically littermates, and we used male and female mice. The number of samples (n) for each comparison can be found in the individual method descriptions and are given in the corresponding figure legends. Phenotyping and data analysis were performed blind to the genotype of the mice. All values are presented either as the mean ± standard deviation (SD) or standard error of the mean (SEM). For statistical analysis, GraphPad Prism software was used. To assess differences in the mean, one-way or two-way ANOVA with Bonferroni's multiple comparison test, and unpaired two-tailed *t*-tests were employed. Significance was determined as *P* < 0.05 (*), *P* < 0.01 (**), *P* < 0.001 (***) or *P* < 0.0001 (****). Complete results of the statistical analyses are included in the figure legends.

## Supplementary Information

**Additional file 1: Fig. S1.** Efficient deletion of ISL1 in the *Isl1CKO* developing pancreas. Representative whole-mount immunolabeling of the pancreas of tdTomato reporter control-*Ai14* and *Isl1CKO-Ai14* embryos during the primary transitions (E10.5 and E11.5) and the secondary transition (E13.5) shows ISL1 expression in the Neurod1Cre positive domain visualized by tdTomato expression. The pancreatic epithelium is delineated by the expression of PDX1. Scale bars: 50 μm. **Fig. S2.** Diabetic phenotype of *Isl1CKO*. **a** The average blood glucose levels over time (from 1 week to 5 weeks of age) in females and males mice fed ad libitum. The 5 weeks of age female mice had blood glucose unmeasurable (above 35 mmol/l), the 5 week of age males show a high variability with 7 mice with blood glucose unmeasurable, and 3 with 0.8, 7.9 and 31.9 mmol/l. Data are presented as mean ± SD, analyzed by VA (****P < 0.0001). **b** The average body weight of adult female and male mice. Data are presented as mean ± SEM, Student’s t test. **c** Glucose tolerance test plotted using glucose vs time in heterozygous (*Neurod1Cre/Isl1flox*^+/−^), and control mice. Data are presented as mean ± SD, analyzed by twoway Anova with Bonferroni post-hoc analysis for glucose vs time. **d** Blood glucose concentration in adults fed ad libitum, 6-8 weeks of age. Only measurable levels of glucose (< 35 mmol/l) are shown for* Isl1CKO*. Data are presented as mean ± SEM, Student’s t test (****P < 0.0001). **e** The weight of pancreas of adult mice. Total pancreas weights of 244 ± 78 mg (n = 22) in adult *Isl1CKO* mutants compared to those of controls (211 ± 57 mg, n = 21). Data are presented as mean ± SEM, Student’s t test. **Fig. S3.** Immunolabeling for the marker of cellular mitosis, phosphorylated histone H3 (pHH3). Representative sections from the control and *Isl1CKO* pancreas immunostained for insulin (INS) and PDX1 (marker of β cells) at P0 and E17.5. Scale bars: 50 μm. **Fig. S4.** H3K4me3 and H3K27me3 CUT&Tag-seq analyses of E14.5 pancreatic endocrine cells. **a** The functional enrichment analysis of the differentially expressed genes in *Isl1CKO* was performed using g: Profiler (Gene Ontology: MF, molecular function; BP, biological processes). **b** A UMAP overview of nine cell clusters of different pancreatic cell types used as a reference for the cell type deconvolution analysis (van Gurp et al., 2019). **c** The deconvolved cell cluster proportions in E14.5 endocrine population from our bulk RNA-seq data of *Isl1CKO* and control. **d** The UCSC browser view of whole genome showing H3K4me3 and H3K27me3 peaks in control and *Isl1CKO* endocrine cells at E14.5 based on CUT&Tag-seq analyses. The mapped read counts distributed across all chromosomes are in comparable read depth for control and *Isl1CKO* samples. **e** Bar plot showing percentage of H3K4me3 and H3K27me3 peaks at promoter regions (± 3 kb from TSS), gene body regions, and intergenic regions. **f** Pie chart illustrating the proportion of differentially expressed genes that differentially exhibited one or both H3K4me3 and H3K27me3 marks at their promoter regions from pairwise comparison of *Isl1CKO* and control pancreatic endocrine cells at E14.5. **Fig. S5.** H3K4me3 and H3K27me3 CUT&Tag-seq analyses of P9 pancreatic endocrine cells. **a** The UCSC browser view of whole genome showing H3K4me3 and H3K27me3 peaks in control and *Isl1CKO* endocrine cells at P9 based on CUT&Tag-seq analyses. The mapped read counts distributed across all chromosomes are in comparable read depth for control and *Isl1CKO* samples. **b** Bar plot showing percentage of H3K4me3 and H3K27me3 peaks at promoter regions (± 3 kb from TSS), gene body regions, and intergenic regions of endocrine cells of the P9 pancreas. **Fig. S6.** ISL1 binding CUT&Tag-seq analyses of E14.5 pancreatic endocrine cells. **a** Pie chart showing genomic distribution of ISL1 loci in E14.5 pancreatic endocrine cells. **b** The most enriched Gene Ontology (GO) biological processes for genes bound by ISL1 and with *Isl1CKO*-specific H3K27me3 depositions. **Fig. S7**. Gating strategy used to isolate tdTomato^+^ cells. Representative example to show gating to purify live and individual tdTomato^+^ cells for RNA-seq and CUT&Tag-seq. **Table S1.** Primer sequences for genotyping. **Table S2.** Primer sequences for RT-qPCR. **Table S3.** List of antibodies.

**Additional file 2: Video S1.** P9 control tdTomato GLP1 INS. Microdissected pancreas of tdTomato reporter control-*Ai14* mice was cleared (CUBIC protocol), immunolabeled, imaged, and reconstructed in 3D using light-sheet fluorescence microscopy (LFSM). Video shows the distribution and formation of islets in the anatomical microenvironment of the pancreas at P9; tdTomato^+^ endocrine cell population (magenta), β cells with expression of insulin (white), and α cells expressing glucagon-like peptide-1 (green).

**Additional file 3: Video S2.** P9 *ISL1CKO* tdTomato GLP1 INS. Microdissected pancreas of tdTomato reporter *Isl1CKO-Ai14* mice were cleared (CUBIC protocol), immunolabeled, imaged, and reconstructed in 3D using light-sheet fluorescence microscopy (LFSM). Video shows the distribution and formation of islets in the anatomical microenvironment of the pancreas; tdTomato^+^ endocrine cell population (magenta), β cells with expression of insulin (white), and α cells expressing glucagon-like (green)

**Additional file 4: Video S3.** P9 control tdTomato GLP1 Tubulin. Microdissected pancreas of tdTomato reporter control-*Ai14* mice were cleared (CUBIC protocol), immunolabeled, imaged, and reconstructed in 3D using light-sheet fluorescence microscopy (LFSM). LFSM video shows the distribution and formation of islets in the anatomical microenvironment of the pancreas at P9; tdTomato^+^ endocrine cells (magenta), α cells expressing glucagon-like peptide-1 (green), and neuronal fibers labeled by antitubulin (white fibers).

**Additional file 5: Video S4.** P9 *Isl1CKO* tdTomato GLP1 Tubulin. Microdissected pancreas of tdTomato reporter *Isl1CKO-Ai14 *mice were cleared (CUBIC protocol), immunolabeled, imaged, and reconstructed in 3D using light-sheet fluorescence microscopy (LFSM). LFSM video shows the distribution and formation of islets in the anatomical microenvironment of the pancreas at P9; tdTomato^+^ endocrine cells (magenta), α cells expressing glucagon-like peptide-1 (green) and neuronal fibers labeled by anti-tubulin (white fibers).

**Additional file 6: Dataset S1a.** Differentially expressed genes based on RNA sequencing of pancreatic endocrine cells at E14.5. **S1b**. Deconvolution analyses. **S1c.** Transcription factor enrichment analysis for differentially expressed genes at E14.5. **S1d.** Differentially expressed genes based on RNA sequencing of P9 pancreatic endocrine cells. **S1e.** GO terms enrichment for differentially expressed genes based on RNA-seq  at P9. **S1f.** Upregulated genes at P9 defining the endocrine progenitor state.

**Additional file 7: Dataset S2.** ISL1 and H3K27me3 CUT&Tag-seq data analyses: pancreatic endocrine cells at E14.5.

**Additional file 8: Dataset S3.** ISL1 and H3K27me3 CUT&Tag-seq data analyses: pancreatic endocrine cells at P9.

## Data Availability

All raw sequence data have been deposited at GEO: https://www.ncbi.nlm.nih.gov/geo. Under accession number GSE206094. All data generated or analyzed during this study are included in this article and its supplemental materials.
