# Supplementary material for: ISL1 controls pancreatic alpha cell fate and beta cell maturation
Source: Cell Biosci. 2023 Mar 10;13:53. doi: 10.1186/s13578-023-01003-9 (PMC9999528; doi:10.1186/s13578-023-01003-9)
Supplement: Supplementary file 1 — Additional file 1: Fig. S1. Efficient deletion of ISL1 in the Isl1CKO developing pancreas. Representative whole-mount immunolabeling of the pancreas of tdTomato reporter control-Ai14 and Isl1CKO-Ai14 embryos during the primary transitions (E10.5 and E11.5) and the secondary transition (E13.5) shows ISL1 expression in the Neurod1Cre positive domain visualized by tdTomato expression. The pancreatic epithelium is delineated by the expression of PDX1. Scale bars: 50 μm. Fig. S2. Diabetic phenotype of Isl1CKO. a The average blood glucose levels over time (from 1 week to 5 weeks of age) in females and males mice fed ad libitum. The 5 weeks of age female mice had blood glucose unmeasurable (above 35 mmol/l), the 5 week of age males show a high variability with 7 mice with blood glucose unmeasurable, and 3 with 0.8, 7.9 and 31.9 mmol/l. Data are presented as mean ± SD, analyzed by VA (****P < 0.0001). b The average body weight of adult female and male mice. Data are presented as mean ± SEM, Student’s t test. c Glucose tolerance test plotted using glucose vs time in heterozygous (Neurod1Cre/Isl1flox+/−), and control mice. Data are presented as mean ± SD, analyzed by twoway Anova with Bonferroni post-hoc analysis for glucose vs time. d Blood glucose concentration in adults fed ad libitum, 6-8 weeks of age. Only measurable levels of glucose (< 35 mmol/l) are shown for Isl1CKO. Data are presented as mean ± SEM, Student’s t test (****P < 0.0001). e The weight of pancreas of adult mice. Total pancreas weights of 244 ± 78 mg (n = 22) in adult Isl1CKO mutants compared to those of controls (211 ± 57 mg, n = 21). Data are presented as mean ± SEM, Student’s t test. Fig. S3. Immunolabeling for the marker of cellular mitosis, phosphorylated histone H3 (pHH3). Representative sections from the control and Isl1CKO pancreas immunostained for insulin (INS) and PDX1 (marker of β cells) at P0 and E17.5. Scale bars: 50 μm. Fig. S4. H3K4me3 and H3K27me3 CUT&Tag-seq analyses of E14.5 pancreatic e [file 13578_2023_1003_MOESM1_ESM.pdf]

# Additional file 1
# Supplemental information

**ISL1 controls pancreatic alpha cell fate and beta cell maturation**

Romana Bohuslavova, Valeria Fabriciova, Laura Lebrón-Mora, Jessica Malfatti, Ondrej Smolik, Lukas Valihrach, Sarka Benesova, Daniel Zucha, Zuzana Berkova, Frantisek Saudek, Sylvia Evans, and Gabriela Pavlinkova

**This PDF file includes:**

Figures S1 to S7
Tables S1 to S3
Legends for Movies S1 to S4

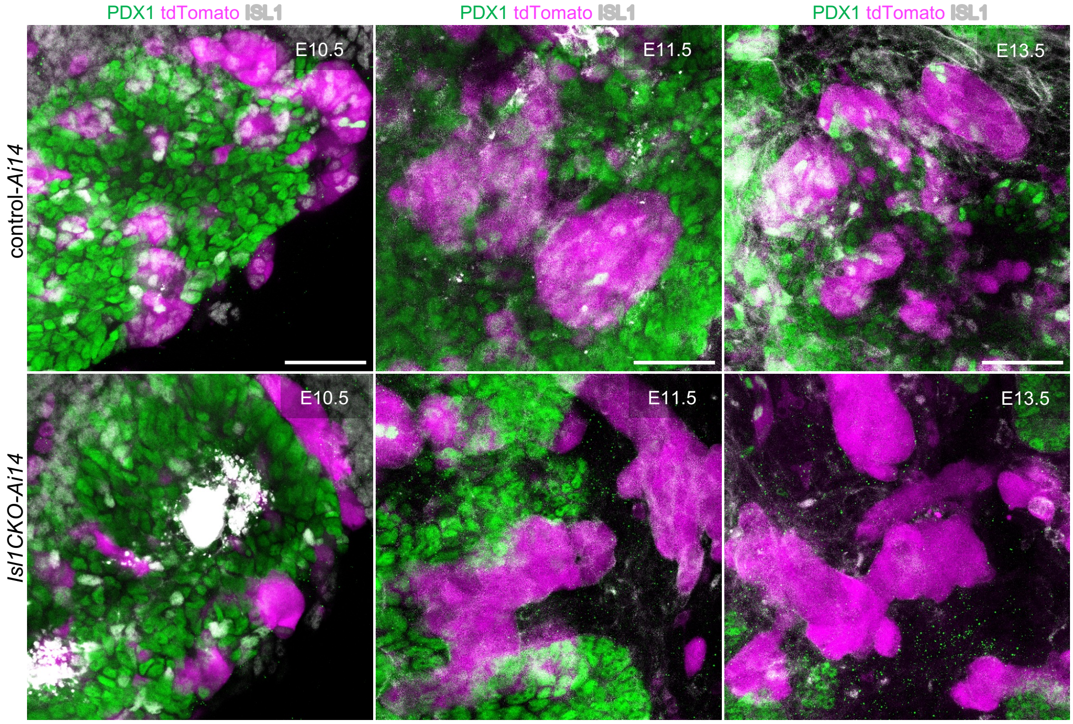


**Fig. S1** Efficient deletion of ISL1 in the *Isl1CKO* developing pancreas. Representative whole-mount immunolabeling of the pancreas of tdTomato reporter control-*Ai14* and *Isl1CKO-Ai14* embryos during the primary transitions (E10.5 and E11.5) and the secondary transition (E13.5) shows ISL1 expression in the Neurod1$^{Cre}$ positive domain visualized by tdTomato expression. The pancreatic epithelium is delineated by the expression of PDX1. Scale bars: 50 µm.

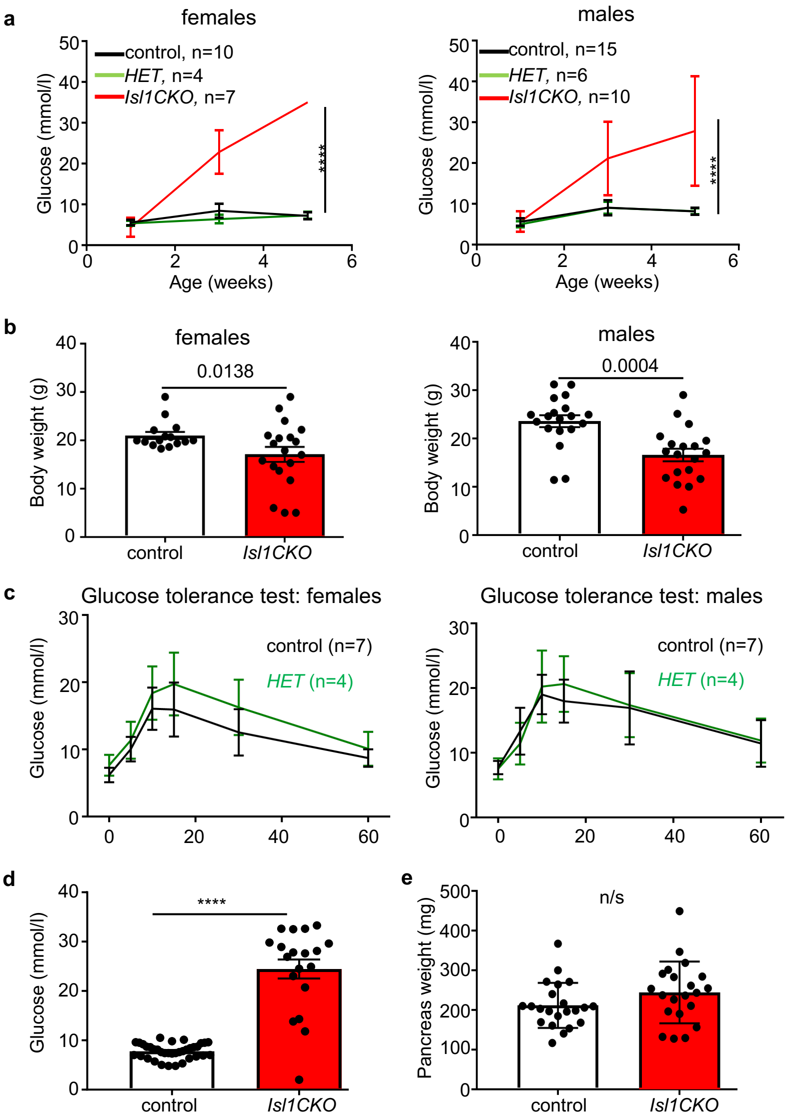


**Fig. S2** Diabetic phenotype of *Isl1CKO*. **a** The average blood glucose levels over time (from 1 week to 5 weeks of age) in females and males mice fed *ad libitum.* The 5 weeks of age female mice had blood glucose unmeasurable (above 35 mmol/l), the 5 week of age males show a high variability with 7 mice with blood glucose unmeasurable, and 3 with 0.8, 7.9 and 31.9 mmol/l. Data are presented as mean ± SD, analyzed by two-way ANOVA (****$P < 0.0001$). **b** The average body weight of adult female and male mice. Data are presented as mean ± SEM, Student's *t* test. **c** Glucose tolerance test plotted using glucose vs time in heterozygous (*Neurod1$^{Cre}$/Isl1flox$^{+/-}$*), and control mice. Data are presented as mean ± SD, analyzed by two-way Anova with Bonferroni post-hoc analysis for glucose vs time. **d** Blood glucose concentration in adults fed *ad libitum*, 6-8 weeks of age. Only measurable levels of glucose (< 35 mmol/l) are shown for *Isl1CKO*. Data are presented as mean ± SEM, Student's *t* test (****$P < 0.0001$). **e** The weight of pancreas of adult mice. Total pancreas weights of 244 ± 78 mg (n = 22) in adult *Isl1CKO* mutants compared to those of controls (211 ± 57 mg, n = 21). Data are presented as mean ± SEM, Student's *t* test.

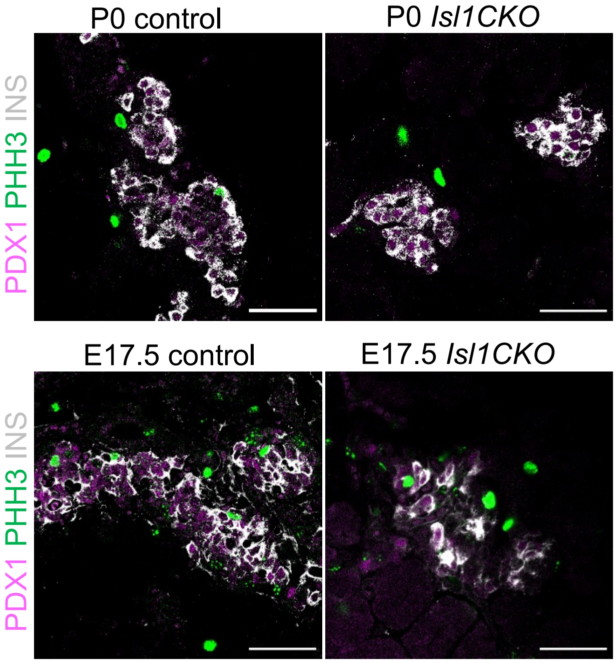


**Fig. S3** Immunolabeling for the marker of cellular mitosis, phosphorylated histone H3 (pHH3). Representative sections from the control and *Isl1CKO* pancreas immunostained for insulin (INS) and PDX1 (marker of β cells) at P0 and E17.5. Scale bars: 50 µm.

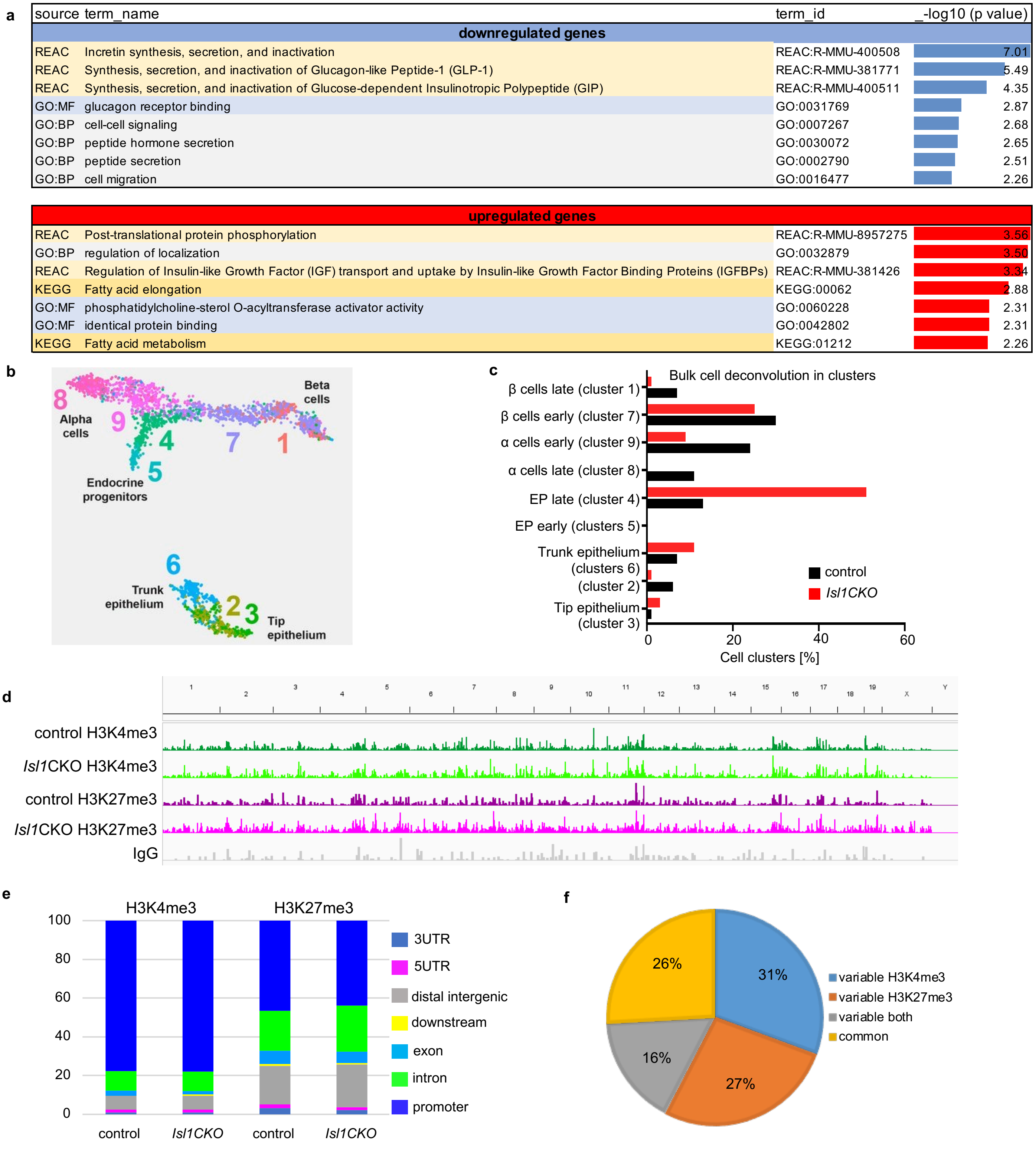


**Fig. S4** H3K4me3 and H3K27me3 CUT&Tag-seq analyses of E14.5 pancreatic endocrine cells. **a** The functional enrichment analysis of the differentially expressed genes in *Isl1CKO* was performed using g: Profiler (Gene Ontology: MF, molecular function; BP, biological processes). **b** A UMAP overview of nine cell clusters of different pancreatic cell types used as a reference for the cell type deconvolution analysis (van Gurp et al., 2019). **c** The deconvolved cell cluster proportions in E14.5 endocrine population from our bulk RNA-seq data of *Isl1CKO* and control. **d** The UCSC browser view of whole genome showing H3K4me3 and H3K27me3 peaks in control and *Isl1CKO* endocrine cells at E14.5 based on CUT&Tag-seq analyses. The mapped read counts distributed across all chromosomes are in comparable read depth for control and *Isl1CKO* samples. **e** Bar plot showing percentage of H3K4me3 and H3K27me3 peaks at promoter regions (± 3 kb from TSS), gene body regions, and intergenic regions. **f** Pie chart illustrating the proportion of differentially expressed genes that differentially exhibited one or both H3K4me3 and H3K27me3 marks at their promoter regions from pairwise comparison of *Isl1CKO* and control pancreatic endocrine cells at E14.5.

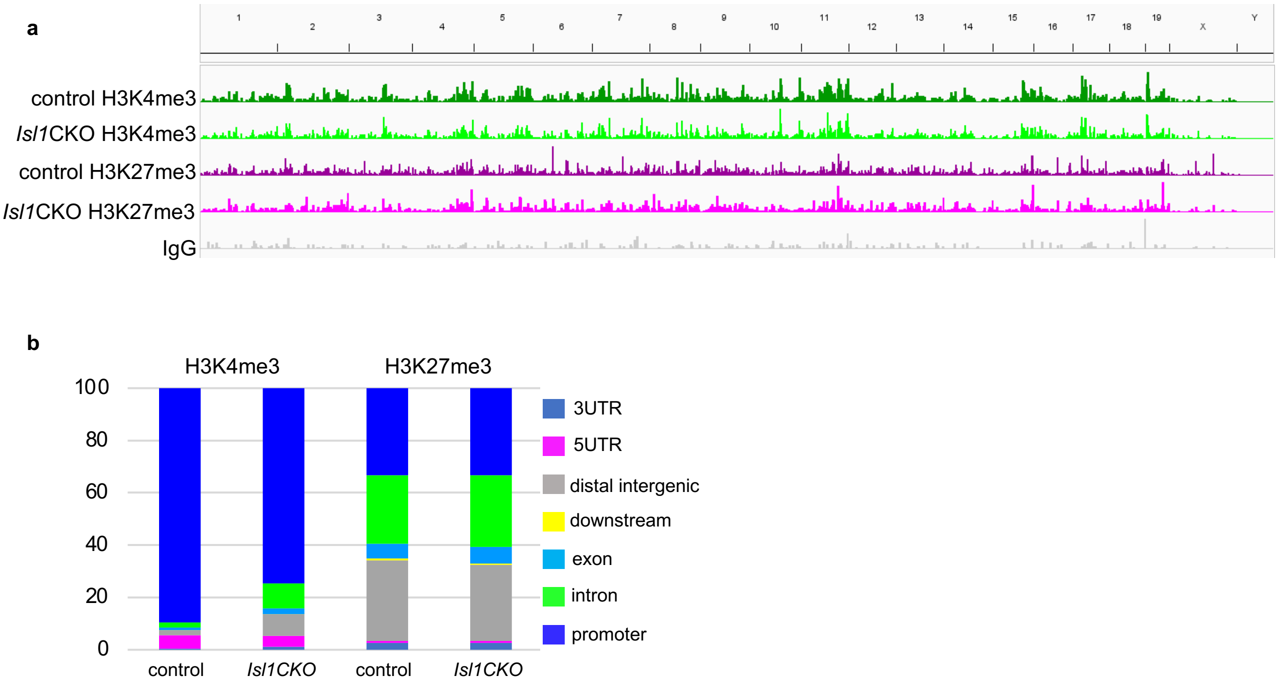


**Fig. S5** H3K4me3 and H3K27me3 CUT&Tag-seq analyses of P9 pancreatic endocrine cells. **a** The UCSC browser view of whole genome showing H3K4me3 and H3K27me3 peaks in control and *Isl1CKO* endocrine cells at P9 based on CUT&Tag-seq analyses. The mapped read counts distributed across all chromosomes are in comparable read depth for control and *Isl1CKO* samples. **b** Bar plot showing percentage of H3K4me3 and H3K27me3 peaks at promoter regions (± 3 kb from TSS), gene body regions, and intergenic regions of endocrine cells of the P9 pancreas.

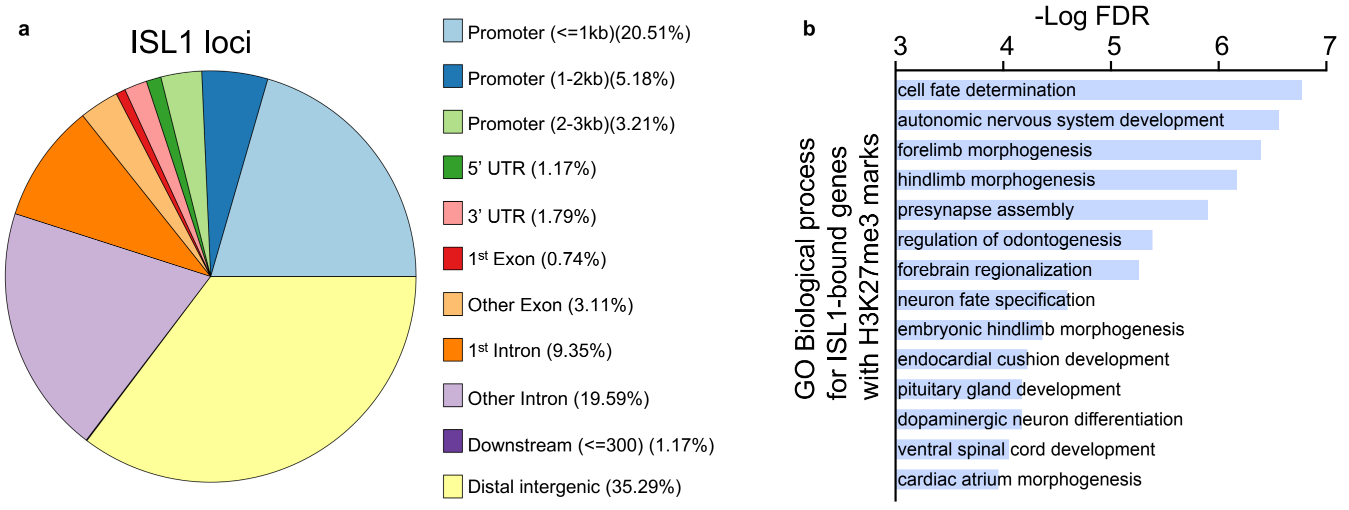


**Fig. S6** ISL1 binding CUT&Tag-seq analyses of E14.5 pancreatic endocrine cells. **a** Pia chart showing genomic distribution of ISL1 loci in E14.5 pancreatic endocrine cells. **b** The most enriched Gene Ontology (GO) biological processes for genes bound by ISL1 and with *Isl1CKO*-specific H3K27me3 depositions.

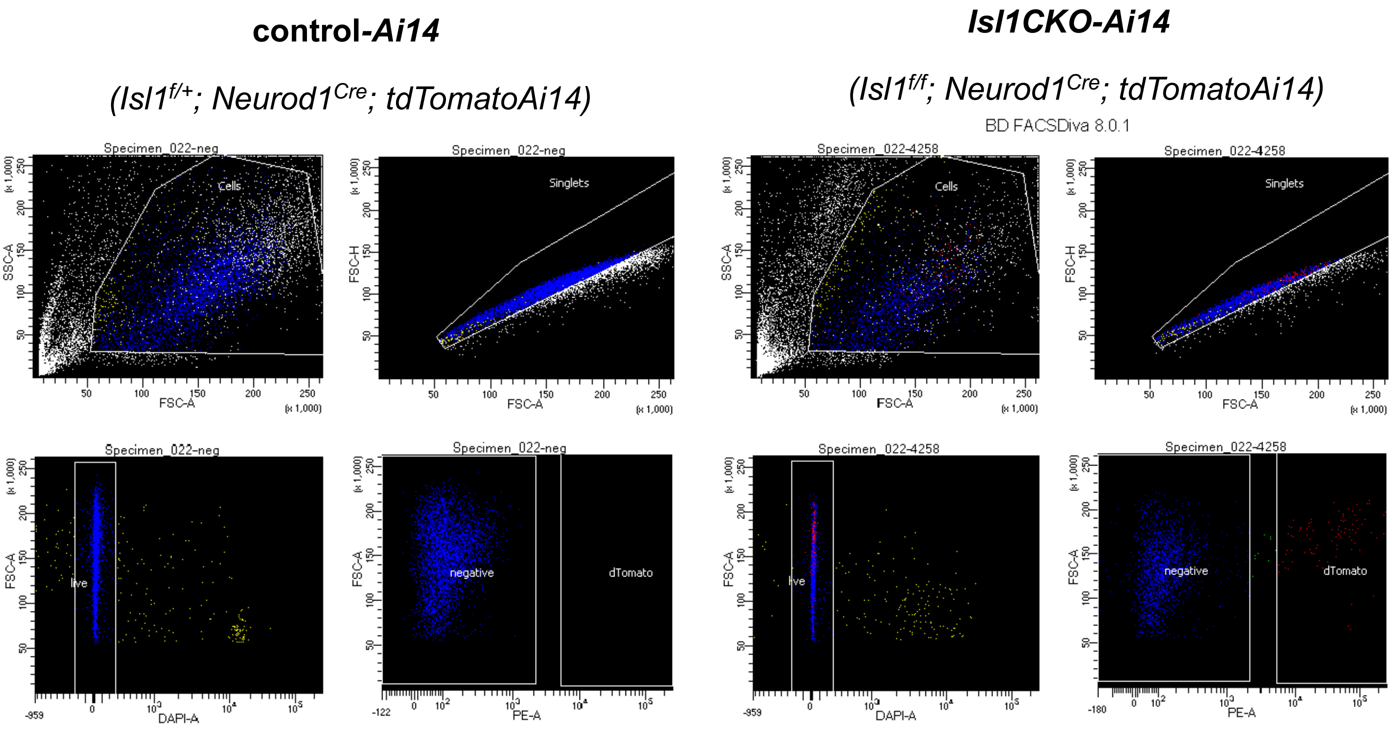


**Fig. S7** Gating strategy used to isolate tdTomato$^{+}$ cells. Representative example to show gating to purify live and individual tdTomato$^{+}$ cells for RNA-seq and CUT&Tag-seq.

## Supplementary Table S1. Primer sequences for genotyping

| Gene | Primer sequence |
|---|---|
| *Isl1 $^{flox}$_F* | 5´ CTCTGGAACATCCCACATTG 3´ |
| *Isl1 $^{flox}$_R* | 5´ GATGCAACCCCTGTTCCTAC 3´ |
| *Neurod1/cre_F* | 5´ CCATTTTGCAGTGGACTCCT 3´ |
| *Neurod1/cre_R* | 5´ ACGGACAGAAGCATTTTCCA 3´ |
| *WT- TomatoAi14_F* | 5´ AAGGGAGCTGCAGTGGAGTA 3´ |
| *WT- TomatoAi14_R* | 5´ CCGAAAATCTGTGGGAAGTC 3´ |
| *Transgene-TomatoAi14_F* | 5´ CTGTTCCTGTACGGCATGG 3´ |
| *Transgene-TomatoAi14_R* | 5´ GGCATTAAAGCAGCGTATCC 3´ |

**Supplementary Table S2. Primer sequences for RT-qPCR**

| Gene | Forward | Reverse |
|---|---|---|
| *Arx* | 5´ TTTTCTAGGAGCAGCGGTGT 3´ | 5´ GGGCCATAGTGGAAAAGAGC 3´ |
| *Fev* | 5´ AAACATCTCTGCCGTTCCCC 3´ | 5´ TGGGAGCTTTAATGGGGCTG 3´ |
| *Gcg* | 5´ CAGAAGAAGTCGCCATTGCC 3´ | 5´ AAGTCCCTGGTGGCAAGATT 3´ |
| *Ghrl* | 5´ CCAGAGGACAGAGGACAAGC 3´ | 5´ ACATCGAAGGGAGCATTGAA 3´ |
| *Ins1* | 5´ GACCAGCTATAATCAGAGACCATC 3´ | 5´ GTAGGAAGTGCACCAACAGG 3´ |
| *Ins2* | 5´ GGCTTCTTCTACACACCCAT 3´ | 5´ CCAAGGTCTGAAGGTCACCT 3´ |
| *Mafb* | 5´ GCAGGTATAAACGCGTCCAG 3´ | 5´ TGAATGAGCTGCGTCTTCTC 3´ |
| *Ngn3* | 5´ AGTGCTCAGTTCCAATTCCAC 3´ | 5´ CGGCTTCTTCGCTTTTTGCTG 3´ |
| *Peg10* | 5´ CCCTCATCCTTCGTGGCATC 3´ | 5´ CTCGTGGTTGGCGTCTTTTG 3´ |
| *Pou3f4* | 5´ CTGCCTCGAATCCCTACAGC 3´ | 5´ CTGCAAGTAGTCACTTTGGAGAA 3´ |
| *Ppy* | 5´ CAGGCGACTATGCGACACC 3´ | 5´ CAGGGAATCAAGCCAACTGG 3´ |
| *Sst* | 5´ ACCGGGAAACAGGAACTGG 3´ | 5´ TTGCTGGGTTCGAGTTGGC 3´ |
| *Hprt1* | 5´ GCTTGCTGGTGAAAAGGACCTCTCGAAG 3´ | 5´ CCTGAAGTACTCATTATAGTCAAGGGCAT 3´ |

## Supplementary Table S3. List of antibodies

| primary antibody | host species | company | catalog number | dilution |
|---|---|---|---|---|
| Alpha-Amylase | rabbit | Sigma-Aldrich | A8273 | 1:2500 |
| Cre Recombinase | rabbit | BioLegend | 908001 | 1:500 |
| GLP1 | rabbit | Abcam | ab111125 | 1:10 000 |
| Glucagon | mouse IgG1 | Abcam | ab10988 | 1:400 |
| Glucagon | goat | BIO-RAD | 4660-1140 | 1:500 |
| Histone H3K4me3 | rabbit | Active Motif | 39159 | 1:100 |
| Histone H3K27me3 | rabbit | Active Motif | 39155 | 1:100 |
| Insulin | rabbit | Cell Signaling | C27C9 | 1:400 |
| Insulin | guinea pig | Abcam | ab7842 | 1:50 |
| *Islet1/2 | mouse IgG2b | Developmental Hybridoma Bank | 39.4D5 | 1:200 |
| Ki67 | rabbit | Cell Signaling | 9129 | 1:400 |
| NeuroD1 | goat | Santa Cruz Biotechnology | sc-1084 | 1:100 |
| NGN3 | mouse | DSHB | F25A1B3 | 1:3 |
| NKX6.1 | mouse | DSHB | F64A6B4 | 1:3 |
| Pax6 | rabbit | BioLegend | 901301 | 1:100 |
| PDX1 | rabbit | Abcam | ab47267 | 1:2 000 |
| PHH3 | rabbit | Merck | 06-570 | 1:100 |
| TUBB3 | mouse | BioLegend | 801202 | 1:500 |

| secondary antibody | host species | company | catalog number | dilution |
|---|---|---|---|---|
| Alexa Fluor® **405**-conjugated AffiniPure Donkey Anti-**Goat** IgG (H+L) | donkey | Jackson ImmunoResearch | 705-475-147 | 1:500 |
| Alexa Fluor® **488**-conjugated AffiniPure Donkey Anti-**Rabbit** IgG (H+L) | donkey | Jackson ImmunoResearch | 711-545-152 | 1:500 |
| Alexa Fluor® **647**-conjugated AffiniPure Donkey Anti-**mouse** IgG | donkey | Jackson ImmunoResearch | 715-605-151 | 1:500 |
| Alexa Fluor® **594** AffiniPure Goat Anti-**Rabbit** IgG (H+L) | goat | Jackson ImmunoResearch | 111-585-144 | 1:500 |
| Alexa Fluor® **488** AffiniPure Goat Anti-**Mouse** IgG (H+L) | goat | Jackson ImmunoResearch | 115-545-146 | 1:500 |
| DyLight **405**-conjugated AffiniPure Goat Anti-**Guinea Pig** IgG (H+L) | goat | Jackson ImmunoResearch | 106-475-003 | 1:500 |

*Monoclonal antibody 39.4D5 was deposited to the DSHB by Jessell, T.M. / Brenner-Morton, S. (DSHB Hybridoma Product 39.4D5).

# Legends for Movies

**Video S1. P9 control tdTomato GLP1 INS**. Microdissected pancreas of tdTomato reporter control-*Ai14* mice was cleared (CUBIC protocol), immunolabeled, imaged, and reconstructed in 3D using light-sheet fluorescence microscopy (LFSM). Video shows the distribution and formation of islets in the anatomical microenvironment of the pancreas at P9; tdTomato$^+$ endocrine cell population (magenta), β cells with expression of insulin (white), and  α cells expressing glucagon-like peptide-1 (green).

**Video S2. P9 ISL1CKO tdTomato GLP1 INS**. Microdissected pancreas of tdTomato reporter *Isl1CKO-Ai14* mice were cleared (CUBIC protocol), immunolabeled, imaged, and reconstructed in 3D using light-sheet fluorescence microscopy (LFSM). Video shows the distribution and formation of islets in the anatomical microenvironment of the pancreas; tdTomato$^+$ endocrine cell population (magenta), β cells with expression of insulin (white), and  α cells expressing glucagon-like peptide-1 (green).

**Video S3. P9 control tdTomato GLP1 Tubulin**. Microdissected pancreas of tdTomato reporter control-*Ai14* mice were cleared (CUBIC protocol), immunolabeled, imaged, and reconstructed in 3D using light-sheet fluorescence microscopy (LFSM). LFSM video shows the distribution and formation of islets in the anatomical microenvironment of the pancreas at P9; tdTomato$^+$ endocrine cells (magenta), α cells expressing glucagon-like peptide-1 (green), and neuronal fibers labeled by anti-tubulin (white fibers).

**Video S4. P9 Isl1CKO tdTomato GLP1 Tubulin.** Microdissected pancreas of tdTomato reporter *Isl1CKO-Ai14* mice were cleared (CUBIC protocol), immunolabeled, imaged, and reconstructed in 3D using light-sheet fluorescence microscopy (LFSM). LFSM video shows the distribution and formation of islets in the anatomical microenvironment of the pancreas at P9; tdTomato$^+$ endocrine cells (magenta), α cells expressing glucagon-like peptide-1 (green) and neuronal fibers labeled by anti-tubulin (white fibers).
